# Systematic review and meta-analysis of hepatitis E seroprevalence in Southeast Asia: a comprehensive assessment of epidemiological patterns

**DOI:** 10.1186/s12879-024-09349-2

**Published:** 2024-05-24

**Authors:** Ulugbek Khudayberdievich Mirzaev, Serge Ouoba, Ko Ko, Zayar Phyo, Chanroth Chhoung, Akuffo Golda Ataa, Aya Sugiyama, Tomoyuki Akita, Junko Tanaka

**Affiliations:** 1 Department of Epidemiology, Infectious Disease Control and Prevention, Graduate School of Biomedical and Health Sciences, Hiroshima University, 1-2-3, Kasumi, Hiroshima, Minami 734-8551 Japan; 2 Department of Hepatology, Research Institute of Virology, Tashkent, Uzbekistan; 3 Unité de Recherche Clinique de Nanoro (URCN), Institut de Recherche en Sciences de La Santé (IRSS), Nanoro, Burkina Faso

**Keywords:** Hepatitis E virus, Prevalence, Southeast Asia, Immunoglobulins, IgM, IgG, Systematic review, Meta-analysis, Epidemiologic patterns

## Abstract

The burden of hepatitis E in Southeast Asia is substantial, influenced by its distinct socio-economic and environmental factors, as well as variations in healthcare systems. The aim of this study was to assess the pooled seroprevalence of hepatitis E across countries within the Southeast Asian region by the UN division.

The study analyzed 66 papers across PubMed, Web of Science, and Scopus databases, encompassing data from of 44,850 individuals focusing on anti-HEV seroprevalence. The investigation spanned nine countries, excluding Brunei and East Timor due to lack of data. The pooled prevalence of anti-HEV IgG was determined to be 21.03%, with the highest prevalence observed in Myanmar (33.46%) and the lowest in Malaysia (5.93%). IgM prevalence was highest in Indonesia (12.43%) and lowest in Malaysia (0.91%). The study stratified populations into high-risk (farm workers, chronic patients) and low-risk groups (general population, blood donors, pregnant women, hospital patients). It revealed a higher IgG—28.9%, IgM—4.42% prevalence in the former group, while the latter group exhibited figures of 17.86% and 3.15%, respectively, indicating occupational and health-related vulnerabilities to HEV.

A temporal analysis (1987–2023), indicated an upward trend in both IgG and IgM prevalence, suggesting an escalating HEV burden.

These findings contribute to a better understanding of HEV seroprevalence in Southeast Asia, shedding light on important public health implications and suggesting directions for further research and intervention strategies.

**Key points**

**Research Question**

Investigate the seroprevalence of hepatitis E virus (HEV) in Southeast Asian countries focusing on different patterns, timelines, and population cohorts.

**Findings**

Sporadic Transmission of IgG and IgM Prevalence:

• Pooled anti-HEV IgG prevalence: 21.03%

• Pooled anti-HEV IgM prevalence: 3.49%

Seroprevalence among specific groups:

High-risk group (farm workers and chronic patients):

• anti-HEV IgG: 28.9%

• anti-HEV IgM: 4.42%

Low-risk group (general population, blood donors, pregnant women, hospital patients):

• anti-HEV IgG: 17.86%

• anti-HEV IgM: 3.15%

Temporal Seroprevalence of HEV:

Anti-HEV IgG prevalence increased over decades (1987–1999; 2000–2010; 2011–2023): 12.47%, 18.43%, 29.17% as an anti-HEV IgM prevalence: 1.92%, 2.44%, 5.27%

**Importance**

Provides a comprehensive overview of HEV seroprevalence in Southeast Asia.

Highlights variation in seroprevalence among different population groups.

Reveals increasing trend in HEV seroprevalence over the years.

Distinguishes between sporadic and epidemic cases for a better understanding of transmission dynamics.

**Supplementary Information:**

The online version contains supplementary material available at 10.1186/s12879-024-09349-2.

## Introduction

Hepatitis E is a major global health concern caused by the hepatitis E virus (HEV), which is a small, nonenveloped, single-stranded, positive-sense RNA virus belonging to the *Paslahepevirus* genus in the *Hepeviridae* family. There are eight genotypes of HEV: HEV-1 and HEV-2 infect only humans, HEV-3, HEV-4, and HEV-7 infect both humans and animals, while HEV-5, HEV-6, and HEV-8 infect only animals [1].

HEV infections affect millions of people worldwide each year, resulting in a significant number of symptomatic cases and deaths. In 2015, the World Health Organization (WHO) reported approximately 44,000 deaths from hepatitis E, accounting for 3.3% of overall mortality attributed to viral hepatitis [2]. The primary mode of transmission for hepatitis E is through the fecal–oral route. Outbreaks of the disease are often associated with heavy rainfall and flooding [3, 4]. Additionally, sporadic cases can occur due to poor sanitation, vertical transmission, blood transfusion or close contact with infected animals, which serve as hosts for the virus [5]. Southeast Asia carries a substantial burden of hepatitis E, influenced by its unique socio-economic and environmental factors as well as variations in healthcare systems. Understanding the seroprevalence of hepatitis E in this region is crucial for implementing targeted public health interventions and allocating resources. To achieve the effective control and prevention of HEV, it is required to address the waterborne transmission and considering the specific characteristics of each region. By taking these measures, healthcare authorities can work towards reducing the global impact of hepatitis E on public health. Systematic reviews and meta-analyses on hepatitis E play a crucial role in synthesizing and integrating existing research findings, providing comprehensive insights into the epidemiology, transmission, and burden of the disease, thereby aiding evidence-based decision-making and public health strategies [6, 7].

Recent systematic reviews and meta-analysis conducted on hepatitis E have varied in their scope or were limited by a smaller number of source materials [8, 9]. The objective of this study was to determine the pooled seroprevalence of hepatitis E in countries within Southeast Asia by aggregating findings from a multitude of primary studies conducted across the region.

## Methods

To commence this systematic review and meta-analysis, we adhered to the Preferred Reporting Items for Systematic Reviews and Meta-Analysis (PRISMA) guidelines and used the PRISMA assessment checklist [Supplementary Table 1]. The study included pertinent research conducted within the population of Southeast Asian countries, as outlined by the United Nations [10], and perform a meta-analysis on the seroprevalence of hepatitis E in this specific region.

### PICOT assessment

#### Population

In this systematic review and meta-analysis, the eligible population comprised individuals from the Southeast Asia region, irrespective of age, gender, ethnic characteristics, or specific chronic diseases. However, studies involving populations outside the designated countries, travelers, migrants, animal species studies, and those lacking clear descriptions of the study population were excluded.

#### Intervention and comparison

Intervention and comparison are not applicable to the prevalence studies.

#### Outcome

Anti-HEV antibodies positivity either total antibodies or IgG or IgM among the Southeast Asian countries' population was assessed.

#### Time frame

All studies conducted between 1987 and 2023 were included in this meta-analysis.

### Search strategy

To conduct the data search, we utilized three databases, namely “PubMed”, “Scopus”, and “Web of Science”. The search terms comprised keywords related to the Hepatitis E virus, such as “Hepatitis E virus” OR “Hepatitis E” OR “HEV” AND names of each country “Brunei”, “Cambodia”, “Timor-Leste” OR “East-Timor”, “Laos” OR “Lao PDR”, “Indonesia”, “Malaysia”, “Myanmar” OR “Burma”, “Philippines”, “Singapore”, “Thailand”, “Vietnam” and “Southeast Asia”.

The search process in the databases finished on May 29^th^, 2023, with two members of the study team conducting independent searches. Subsequently, the search results were unified. A grey literature search was performed from June 25^th^ to 30^th^, 2023, by examining the references of review manuscripts and conference materials, along with using specific keywords in the Google Scholar database. Notably, during the gray literature search, additional studies from the Philippines that were initially missing in the first search were identified and included. Moreover, due to the diverse language expertise of the team, studies in Russian and French related to Cambodia and Vietnam were also considered for inclusion.

After applying the inclusion and exclusion criteria, each article selected for this systematic review (SR) was considered relevant. The quality assessment of each article was conducted using specific JBI critical appraisal instruments [11] [Supplementary Table 2].

### Sporadic transmission of HEV infection

For the systematic review and meta-analysis of sporadic infection of HEV, we divided the study population into cohorts by countries, by risk of acquiring HEV—low and high risk. The low risk cohort included the general population (apparently healthy individuals, students, some ethnic populations, or individuals included in original studies as “general population”), blood donors, pregnant women, and hospital patients, while pig farmers, those with chronic hepatitis, HIV positive patients, and solid organ transplant patients in the high-risk group.

Lastly, we analyzed data in three decades—1987–1999, 2000–2010, and 2011–2023—to reveal seroprevalence rates over time.

### Epidemic outbreaks of HEV infection

We separated epidemic outbreaks from sporadic cases due to distinct patterns and scale of transmission in epidemy. Epidemics are characterized by rapid and widespread transmission, affecting a large population within a short period and often following a specific pattern or route of propagation.

### Statistical analysis

A meta-analysis of proportions was conducted using the 'meta' and 'metafor' packages in the R statistical software. To account for small proportions, the Freeman-Tukey double arcsine method was applied to transform the data. The Dersimonian and Laird method, which employs a random-effects model, was utilized for the meta-analysis, and the results were presented in a forest plot. Confidence intervals (CIs) for the proportions of individual studies were computed using the Clopper-Pearson method.

Heterogeneity was evaluated using the Cochran Q test and quantified by the I^2^ index. Heterogeneity was considered significant if the *p*-value of the Cochran Q test was below 0.05.

For the assessment of publication bias, a funnel plot displaying the transformed proportions against the sample size was created. The symmetry of the plot was examined using the Egger test (*p* < 0.1).

## Results

**Fig. 1:**
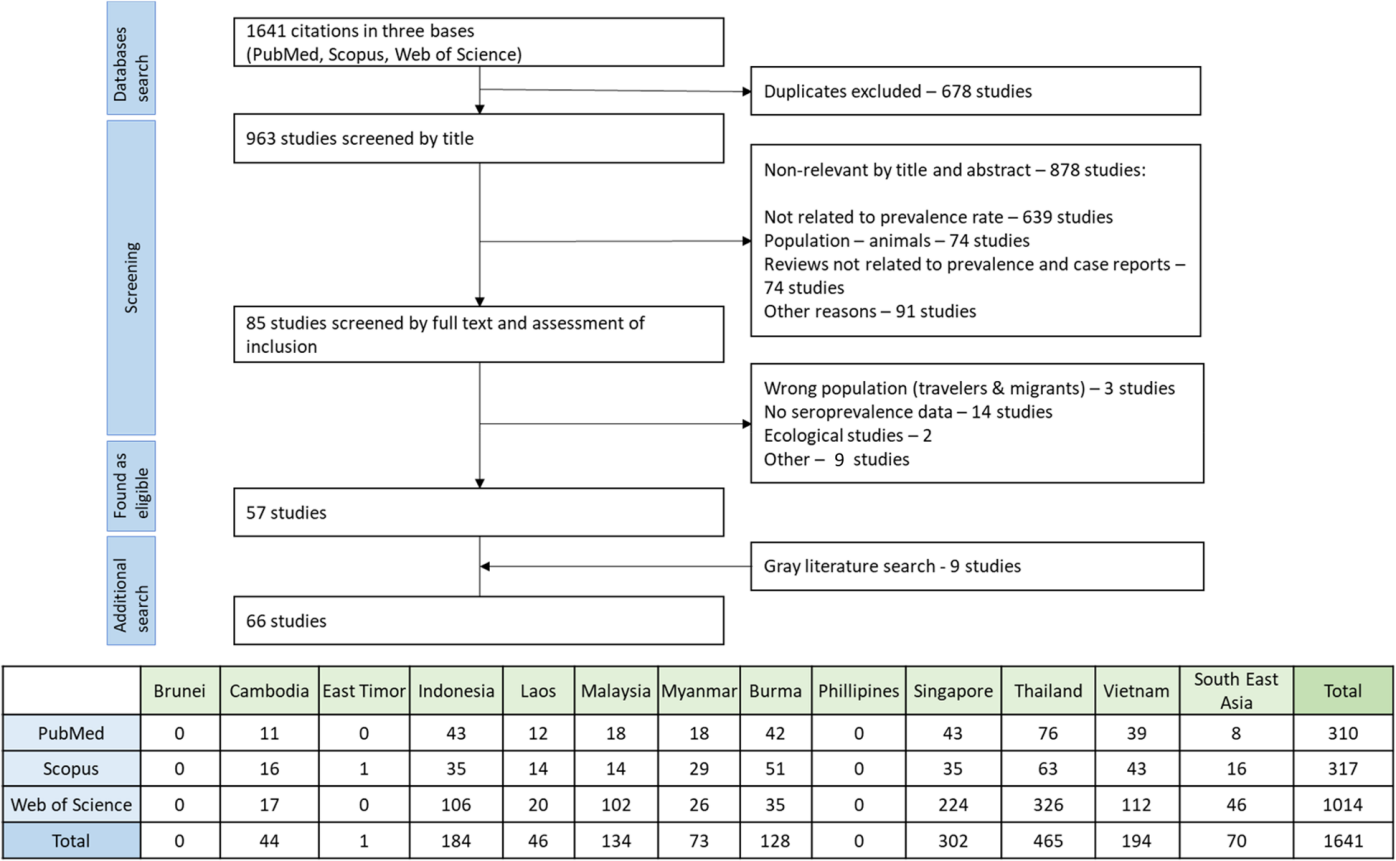
Flowchart of the identification, inclusion, and exclusion of the study. Table under flowchart informing about the studies which were found by the initial search in databases

The initial search yielded 1641 articles, which covered 9 out of 11 Southeast Asia countries. We couldn't find any information on hepatitis E from Brunei. We excluded a study from East Timor because it focused on the wrong population (US Army troops). The final screening resulted in the selection of 57 relevant studies, and the grey literature search added 9 more papers that met our inclusion criteria (Fig. 1). Among 9 papers through a grey literature, two relevant studies from the Philippines [12, 13], one each from Indonesia [14] and Lao PDR [15], one study covered both Vietnam and Cambodia [16], one study provided HEV seroepidemiology information for Myanmar, Thailand, and Vietnam [17], two studies reported in Russian [18, 19] (from Vietnam) and one reported in French [16] (from Vietnam and Cambodia). In total, our analysis included 66 papers from which we extracted data. This involved a total of 44,850 individuals (Table 1).

**Table 1:** Characteristics of included studies reporting HEV seroprevalence in Southeast Asian countries

Study	Study design	Sampling method	Single/ Multicenter	Cities, provinces	Study population	Assay immunoglobulins	Immunoassay	Reference
**Cambodia**								
Kasper M, 2012	CS	Con	M	Around Phnom Penh	HP	IgG, IgM	MP Biomedicals 3.0	[20]
Nouhin J, 2018	ROS	Con	S	Several provinces of Cambodia	GP	IgG, IgM	Wantai Bio-Pharma	[21]
Nouhin J, 2016	CS	Con	S	Phnom Penh	BD	IgG, IgM	Wantai Bio-Pharma	[22]
Nouhin J, 2015	CS	Con	S	Phnom Penh	HP, CP	IgG, IgM	Wantai Bio-Pharma	[23]
Yamada H, 2015	CS	Con	S	Siem Reap	GP	IgG, IgM	Institute of Immunology	[24]
Chhour Y, 2002	CS	Con	S	Phnom Penh	HP	IgG, IgM	WRAIR	[25]
Buchy P, 2004	CS	Con	S	Phnom Penh	HP	IgG	Abbot Laboratories	[16]
**Indonesia**								
Utsumi T, 2011	CS	Con	M	Java, Bali	GP, FW	IgG	Institute of Immunology & MP Diagnostics 4.0	[26]
Achwan W, 2007	CS	Con	M	Tahuna	GP	IgG	ELISA by Mizuo et al. [27]	[28]
Surya I, 2005	CS	Con	M	Bali	PW	Total Ig	ELISA by Mizuo et al. [27]	[29]
Wibawa I, 2007	CS	Con	S	Bali	HP	IgG, IgM	ELISA by Mizuo et al. [27]	[30]
Sedyaningsih-Mamahit E, 2002	CS	Con	M	Bondowoso, East Java	O	IgG, IgM	AFRIMS ELISA	[31]
Widasari D, 2013	CS	Con	M	Java and Bali	GP, FW	IgG	MP Diagnostics 4.0	[32]
Corwin A, 1997	CS	Con	M	West Kalimantan	O	IgG	Abbot Laboratories	[33]
Corwin A, 1995	CS	Con	M	West Kalimantan	O	IgG	Genelab Diagnostics	[34]
Wibawa ID, 2004	CS	Con	M	Bali, Lombok, Surabaya	GP, BD	IgG	ELISA by Mizuo et al. [27]	[35]
Jennings G, 1994	CS	Con	NA	West Kalimantan	HP	IgG, IgM	ELISA by Goldsmith et al. [36]	[14]
**Lao PDR**								
Bounlu K, 1998	CCS	Ran	M	Vientiane	HP	IgG, IgM	Abbot Laboratories & Genelab diagnostics	[37]
Khounvisith V, 2023	CS	Con	S	Vientiane	CP, HP	Total Ig	Diasorin	[38]
Khounvisith V, 2018	CS	Con	M	Vientiane	BD, FW	IgG	Euroimmun	[39]
Tritz S, 2018	CCS	Con	M	Xanthany district of Vientiane	FW, GP	IgG	Abia HEV IgM/IgG	[40]
Bisayher S, 2019	CS	Ran	M	Xieng Khouang province	PW	IgG	EIAgen HevAb	[41]
Holt H, 2016	CS	Ran	M	Two provinces	GP	Total Ig	MP Diagnostics	[42]
Syhavong B, 2010	CS	Con	M	Vientiane	HP	IgG	AFRIMS ELISA	[43]
Chansamouth V, 2016	Co-S	Con	M	Vientiane	PW	IgG, IgM	Wantai Bio-Pharma	[44]
Pauly A, 2016	CS	Con	M	NA	FW	Total Ig	NA	[15]
**Malaysia**								
Wong L, 2022	CS	Con	M	Peninsular Malaysia	FW	IgG, IgM	Wantai Bio-Pharma	[45]
Wong L, 2022	CS	Con	M	Klang Walley	BD	IgG, IgM	Wantai Bio-Pharma	[46]
Wong L, 2020	CS	Con	M	Negeri Sembilan, Selangor	GP	IgG, IgM	Wantai Bio-Pharma	[47]
Ng K, 2000	CS	Con	M	Kuala Lumpur, Klang Valley, Kajang	CP	IgG	Abbot Laboratories	[48]
Hudu, S, 2018	CS	Con	S	Selangor Darul Ehsan	CP	IgG	Wantai Bio-Pharma	[49]
Seow H, 1999	CS	Con	M	Betau-Pahang, Parit Tanjung-Perak and Kuala-Lumpur	GP, BD	IgG, IgM	ELISA by Anderson et al. [50]	[51]
Saat Z, 1999	CS	Con	M	Kelantan and Terengganu	HP	IgG, IgM	Genelab Diagnostics	[52]
**Myanmar**								
Abe K, 2006	CS	Con	NA	Yangon	CP	IgG	ELISA by Li et al. [53]	[17]
Uchida T, 1993	CS	Con	S	Yangon	O	Total Ig	Developed by authors	[54]
Nakai K, 2001	CS	Con	S	Yangon	CP	IgG, IgM	ELISA by Li et al. [53]	[55]
**Philippines**								
Lorenzo A, 2015	CS	Con	S	Manila	BD	IgG, IgM	GenWay Biotechnologies	[13]
Gloriani-Barzaga N, 1997	CS	Con	S	Manila	HP	IgG, IgM	Genelab Diagnostics	[12]
**Singapore**								
Chow W, 1996	CS	Ran	S	Singapore	HP, CP	IgG	Genelab diagnostics	[56]
Wong C, 2019	CS	Con	M	Singapore	GP	IgG, IgM	MP Diagnostics & Mikrogen	[57]
Tan L, 2013	ROS	Con	M	Singapore	HP	IgG, IgM	MP Diagnostics 3.0	[58]
**Thailand**								
Pourpongporn P, 2009	CS	Con	S	Nakorn-Nayok, nearby provinces	FW, GP	IgG	Genelab Diagnostics	[59]
Siripanyaphinyo U, 2014	ROS	Con	M	Bangkok, Krung Thep Maha Nakhon	HP	IgG, IgM	DIA.PRO	[60]
Poovorawan Y, 2016	CS	Con	M	Bangkok, Khonkan, Nakornrajasrima, Nakornsrithamrat	PW, CP, HP, GP	IgG	Genelab Diagnostics	[61]
Maneerat Y, 1996	CS	Con	M	Several hospitals	HP	IgG, IgM	Genelab Diagnostics	[62]
Sa-nguanmoo P, 2015	CS	Ran	M	Lob Buri and Narathiwat	GP	IgG	Euroimmun	[63]
Pilakasiri C, 2009	CS	Con	S	Bangkok	GP	Total Ig	WRAIR ELISA	[64]
Louisirirotchanakul S, 2002	CS	Ran	M	Northern Thailand	GP	Total Ig	Indirect In-house ELISA	[65]
Jupattanasin S, 2019	CS	Con	M	Provinces of Thailand	BD	IgG	Euroimmun	[66]
Hinjoy S, 2013	CS	Con	M	Nan province	FW, GP	IgG	Indirect In-house ELISA	[67]
Getsuwan S, 2023	CS	Con	S	Bangkok	CP	IgG, IgM	Euroimmun	[68]
Gonwong S, 2014	CS	Ran	NA	Provinces Thailand	GP	IgG	DIA.PRO	[69]
Komolmit P, 2020	Co-S	Con	S	Bangkok	CP	IgG, IgM	Wantai Bio-Pharma	[70]
Jutavijittum, P., 2000	CS	Con	M	Chiang Mai, Chiang Rai, Lampang and Lamphun provinces	BD	Total Ig	Anogen	[71]
Boonyai A, 2021	ROS	Con	S	Bangkok	HP, PW, CP	IgG, IgM	Euroimmun	[72]
Abe K, 2006	CS	Con	M	Bangkok	CP	IgG	ELISA by Li et al. [53]	[17]
**Vietnam**								
Huy P, 2021	CS	Con	S	Tra Vinh Province	PW	IgG, IgM	Wantai Bio-Pharma	[73]
Ostankova Y, 2021	CS	Con	M	South Vietnam Provinces	GP, CP	IgG, IgM	DS IFA-ANTI-HEV-G/M	[19]
Hoan N, 2019	CS	Con	M	Hanoi	GP, FW	IgG, IgM	MP Diagnostics	[74]
Hoan N, 2015	CS	Con	M	Hanoi	GP, CP	IgG, IgM	MP Diagnostics	[75]
Lichnaia E, 2021	CS	Con	M	Ha Giang	GP	IgG	DS IFA-ANT I-HEV-G/M	[18]
Hau C, 1999	CS	Con	M	An Giang	GP	IgG	Abbot Laboratories	[76]
Corwin A, 1996	CCS	Con	S	Hanoi	GP, HP	IgG, IgM	Abbot Laboratories & Genelab diagnostics	[77]
Corwin A, 1996	CS	Con	M	An Phu	O	IgG, IgM	Abbot Laboratories & Genelab diagnostics	[78]
Berto A, 2018	CS	Con	M	Dong Thap	FW, HP	IgG	Wantai Bio-Pharma	[79]
Abe K, 2006	CS	Con	NA	Hanoi and Ho Chi Min	CP	IgG	ELISA by Li et al. [53]	[17]
Shimizu K, 2016	CS	Con	S	Hanoi	HP	IgG	ELISA by Li et al. [80]	[81]
Nghiem X, 2018	CS	Con	NA	Northern Vietnam	GP, FW	IgG, IgM	NA	[82]
Tran H, 2003	CS	Con	M	Ho Chi Min	CP	IgG, IgM	ELISA by Li et al. [53]	[83]
Buchy P, 2004 (42)	CS	Con	S	Ho Chi Min	HP	IgG	Abbot Laboratories	[16]

Study design: *CS* cross-sectional, *CCS* case–control study, *Co-S* cohort study, *ROS* retrospective observational study

Sampling method: *Con.* Convenient, *Ran*. random

Multi/single center study: *M* multicenter, *S* single center, *NA* not applicable

Study population: *BD* blood donors, *CP* chronic patients (patients with chronic liver disease, HIV, or solid organ transplant), *FW* pig farm workers, abattoirs, slaughterhouse workers, *GP* general population, *HP* hospital patients with acute liver disorder, *O* epidemic outbreak, *PW* pregnant women

Assay immunoglobulins: *Total Ig* anti-HEV total immunoglobulins, *IgG* anti-HEV immunoglobulin G, *IgM* anti-HEV immunoglobulin M

Immunoassay: *AFRIMS ELISA* Armed Forces Research Institute of Medical Sciences ELISA, *WRAIR ELISA* Walter Reed Army Institute of Research ELISA

### Sporadic transmission IgG and IgM prevalence in Southeast Asian countries (excluding outbreak settings)

**Fig. 2:**
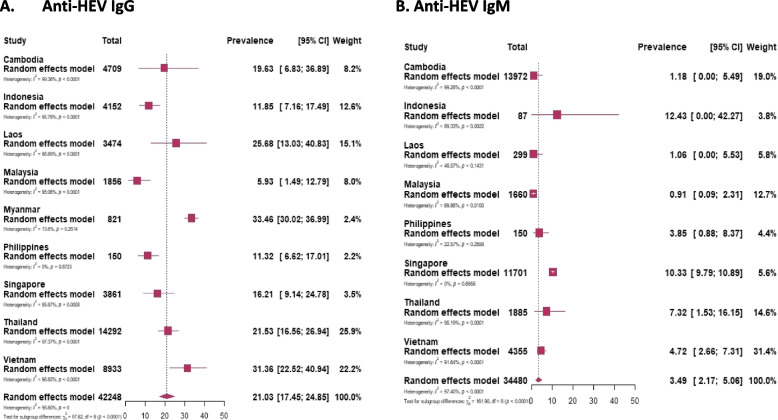
Forest plot of meta-analysis of the prevalence of anti-HEV IgG (**A**) and anti-HEV IgM (**B**) in Southeast Asian countries. The plot includes the number of study participants for each country

The sporadic cases involving 42,248 participants out of 44,850 participants (the remaining 2,602 people are considered in the “Epidemic outbreaks” section) from Southeast Asian countries the pooled prevalence of IgG was found to be 21.03%, while for IgM, it was 3.49% among 34,480 individuals who were tested (Fig. 2). Among these countries, Myanmar registered the highest pooled prevalence of IgG at 33.46%, while Malaysia had the lowest at 5.93%. For IgM prevalence, Indonesia had the highest rate at 12.43%, and Malaysia again had the lowest at 0.91% (Table 2) [Supplementary Figures 1 and 6].

**Table 2:** Prevalence of immunoglobulins (Ig total, IgM, IgG) included studies by countries, specific populations, and groups

	**Anti-HEV IgG**					**Anti-HEV IgM**				
**Subjects**	**Number of studies**	**Prevalence (%)**	**95% CI**	**I**^**2**^	**Cochrane Q-test *p*-value**	**Number of studies**	**Prevalence** **(%)**	**95% CI**	**I**^**2**^	**Cochrane Q-test *p*-value**
***By country***										
**Cambodia**	7	19.63	6.8–36.9	99.4%	< 0.001	7	1.18	0–5.5	99.3%	< 0.001
**Indonesia**	11	11.85	7.2–17.5	95.8%	< 0.001	2	12.43	0- 42.3	89.3%	0.002
**Lao PDR**	13	25.68	13–40.8	98.9%	< 0.001	3	1.06	0–5.5	48.6%	0.14
**Malaysia**	7	5.93	1.5–12.8	95.1%	< 0.001	5	0.91	0.09–2.3	69.9%	0.01
**Myanmar**	2	33.46	30–37	13.8%	0.28	1	1.08	0.23–2.4	-	-
**Philippines**	2	11.32	6.6–17	0.0%	0.87	2	3.85	0.9–8.4	22.6%	0.25
**Singapore**	3	16.21	9.1–24.8	85.9%	< 0.001	2	10.33	9.8–10.9	0.0%	0.69
**Thailand**	23	21.53	16.6–26.9	97.4%	< 0.001	7	7.32	1.5–16.15	95.2%	< 0.001
**Vietnam**	19	31.36	22.5–40.9	98.9%	< 0.001	12	4.72	2.7–7.3	91.6%	< 0.001
***By population group***										
**Blood donors**	10	11.8	5.9–19.3	98.0%	< 0.001	3	0.83	0.37–1.4	0.0%	0.42
**Chronic patients**	16	29.2	22.1–36.9	96.5%	< 0.001	9	3.89	0.98–8.3	95.0%	< 0.001
**Farm workers**	11	28.4	16.6–41.9	97.6%	< 0.001	3	6.2	0.67–16.1	92.3%	< 0.001
**General population**	25	21.4	14.8–28.8	99.3%	< 0.001	9	2.62	0.43–6.42	98.4%	< 0.001
**Hospital patients**	19	16.3	9.1–25.0	98.5%	< 0.001	14	4.44	2.16–7.36	97.4%	< 0.001
**Pregnant women**	6	18.6	11.8–26.4	91.1%	< 0.001	3	1.54	0–5.26	65.3%	0.056
***By HEV acquiring risk***										
**High-risk**	27	28.9	22.7–35.5	97%	< 0.001	11	4.42	1.7–8.2	94.3%	< 0.001
**Low-risk**	60	17.86	13.9–22.2	99%	< 0.001	29	3.15	1.7–5.0	97.9%	< 0.001
***Outbreak assessment (total Ig)***										
**Outbreaks**	5	61.59	57–66	67.80	0.01					

*I*^2^ the percentage of variation of heterogeneity across studies, *95% CI* the 95% confidence interval (level)

### Seroprevalence among specific groups

#### High risk of acquiring HEV

The high-risk group, which included farm workers and chronic patients, demonstrated a pooled anti-HEV IgG prevalence of 28.9%, with IgM prevalence at 4.42% [Supplementary Figures 2 and 8].

#### Chronic patients

This group, comprising individuals with chronic liver disease, HIV infection, or solid organ transplantation, exhibited the highest prevalence of pooled IgG among all cohorts, standing at 29.2%. Additionally, IgM prevalence was 3.9% [Supplementary Figures 2 and 7].

#### Farm workers

Farm workers were divided into several subgroups based on exposure to animals (reservoirs of HEV), including pig or ruminant farmers, slaughterhouse workers, butchers, and meat retailers. Among this group, the highest IgG prevalence was observed at 28.4%, while the pooled IgM level was 6.21% [Supplementary Figures 2 and 7].

#### Low risk of acquiring HEV

The low-risk group, comprising the general population, blood donors, pregnant women, and hospital patients, exhibited anti-HEV IgG and IgM prevalence of 17.86% and 3.15%, respectively. [Supplementary Figures 2 and 9].

#### General population

The general population in Southeast Asian countries, represented by 22,571 individuals, showed a presence of IgG in 21.4% of them. IgM was tested in 10,304 participants, and 2.63% of acute infection cases were identified [Supplementary Figures 2 and 7].

#### Blood donors

Blood donors, as a selected subgroup of the general population, exhibit differences in health status, age, gender distribution, and representativeness, warranting separate assessment. Among blood donors in Southeast Asian countries, the pooled prevalence of IgG and IgM were found to be 11.77% and 0.83%, respectively [Supplementary Figures 2 and 7].

#### Pregnant women

Pregnant women considered a vulnerable group regarding disease consequences, demonstrated an anti-HEV IgG prevalence of 18.56% among 1,670 individuals included in the study. Furthermore, 1.54% of them tested positive for anti-HEV IgM [Supplementary Figures 2 and 7].

#### Hospital patients

A group of 18,792 patients who visited hospitals with clinical signs of acute infection, jaundice, high temperature, and elevated liver enzymes, showed anti-HEV IgG and IgM prevalence of 16.3% and 4.45%, respectively [Supplementary Figures 2 and 7].

### Temporal seroprevalence of HEV

**Fig. 3:**
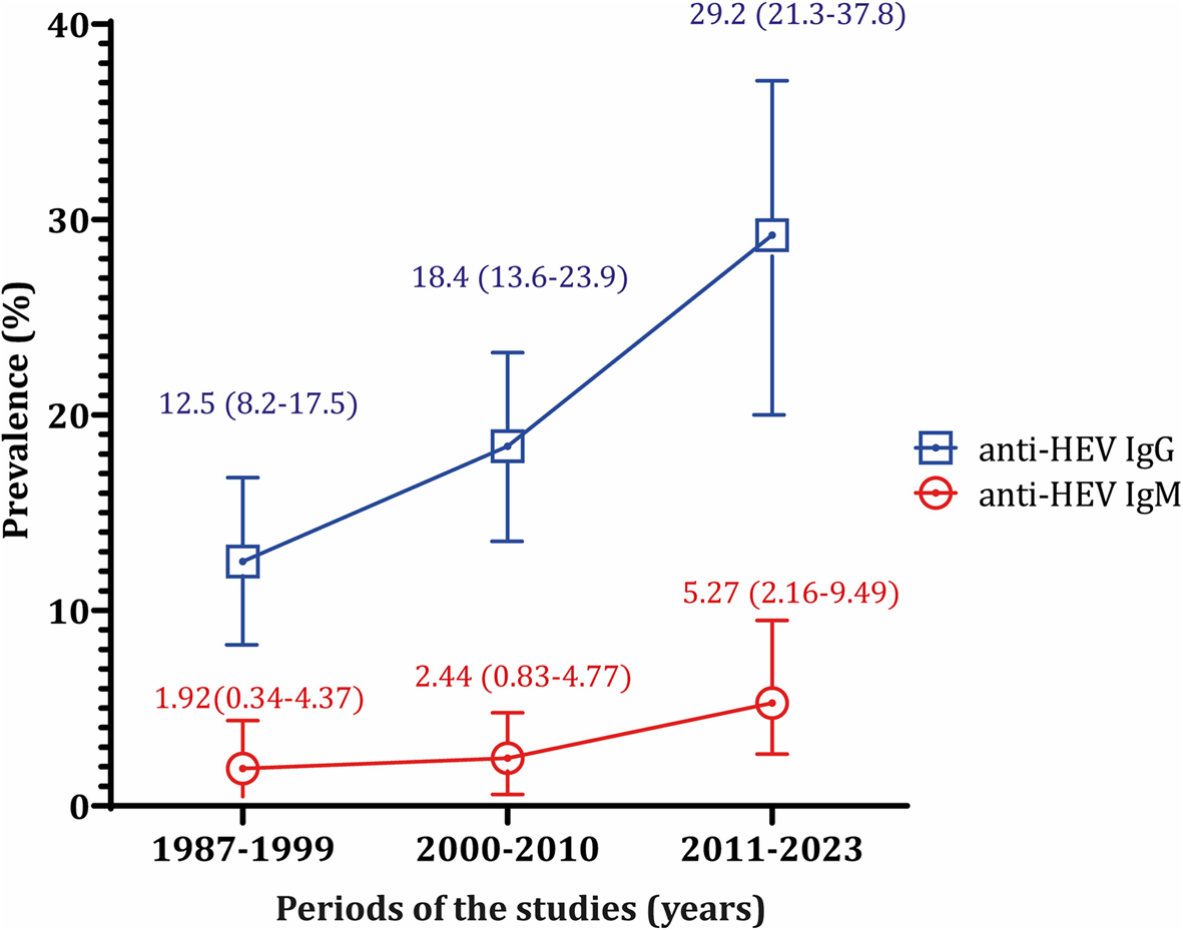
The prevalence of anti-HEV IgG and IgM in Southeast Asian countries throughout the decades

Given the studies' long duration, the data was presented by decades: 1987–1999, 2000–2010, and 2011–2023. The prevalence of IgG showed an upward trend over these decades, with rates of 12.47%, 18.43%, and 29.17%. Similarly, for IgM, the prevalence rates were 1.92%, 2.44%, and 5.27% for the first, second, and third decades, respectively (Fig. 3).

Evaluating the trend of seroprevalence over decades within the same population and country proved challenging due to the limited availability of research papers. Consequently, we assessed anti-HEV antibody prevalence over decades, considering population cohorts and individual countries.

**Fig. 4:**
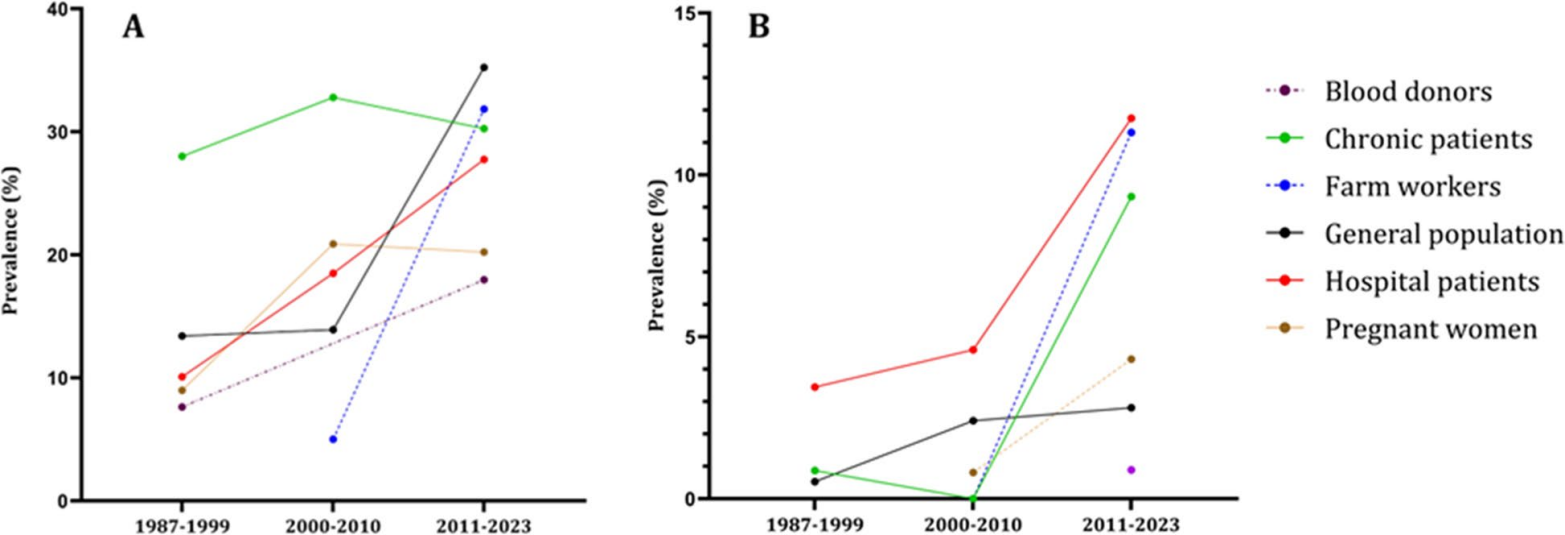
The epidemiological data regarding the occurrence of anti-HEV IgG (**A**) and anti-HEV IgM (**B**) antibodies within population cohorts across Southeast Asian nations divided by decades. The population cohorts delineated by the disrupted lines in the figure lack comprehensive data representation, as they provide information for only two out of three decades. Blood donors group has the anti-HEV IgM only for the last decade

**Fig. 5:**
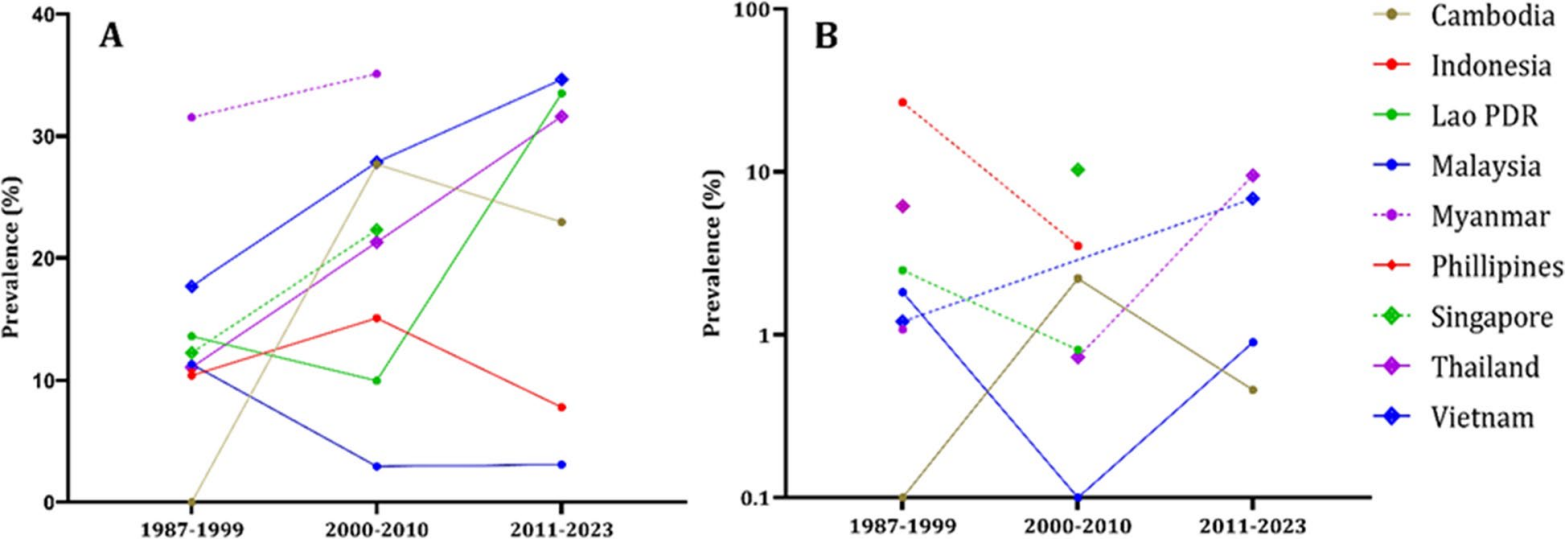
The epidemiological data regarding the occurrence of anti-HEV IgG (**A**) and anti-HEV IgM (**B**) antibodies within countries of Southeast Asia divided by decades. The countries delineated by the disrupted lines in the figure lack comprehensive data representation, as they provide information for only two out of three decades. Philippines has the anti-HEV IgG antibodies information only for the first decade. Philippines, Myanmar, Singapore have anti-HEV IgM information only for single decade

In Fig. 4, we can see that all population groups show a consistent increase in the prevalence of both IgG and IgM antibodies over the decades. Figure 5, we analyze the prevalence of anti-HEV antibodies in different countries over time, except for Indonesia and Malaysia, where we observe an increase in prevalence.

Some studies lacked information on the collection time of the samples [13, 19, 41, 48, 59, 62, 64, 82]. In these studies, the pooled IgG and IgM prevalence was 26.5% and 4.75%, respectively [Supplementary Figures 3, 4, 5, 10, 11, 12].

### Epidemic outbreaks

We separated epidemic outbreaks from sporadic cases due to distinct patterns and scale of transmission in epidemy. Epidemics are characterized by rapid and widespread transmission, affecting a large population within a short period and often following a specific pattern or route of propagation. The outbreaks occurred between 1987 and 1998 in several Southeast Asian countries, namely Indonesia [31, 33, 34], Vietnam [77], and Myanmar [54] [Supplementary Figure 13]. These outbreak investigations involved a total of 2,602 individuals, with most participants from Indonesia (2,292 individuals). The studies were mainly conducted using a case–control design. Among the participants, 876 were considered controls, while 1,726 were classified as cases. The pooled prevalence of total anti-HEV immunoglobulins was estimated as 61.6% (95% CI 57.1–66) (Table 2).

### Assessment of publication bias

**Fig. 6:**
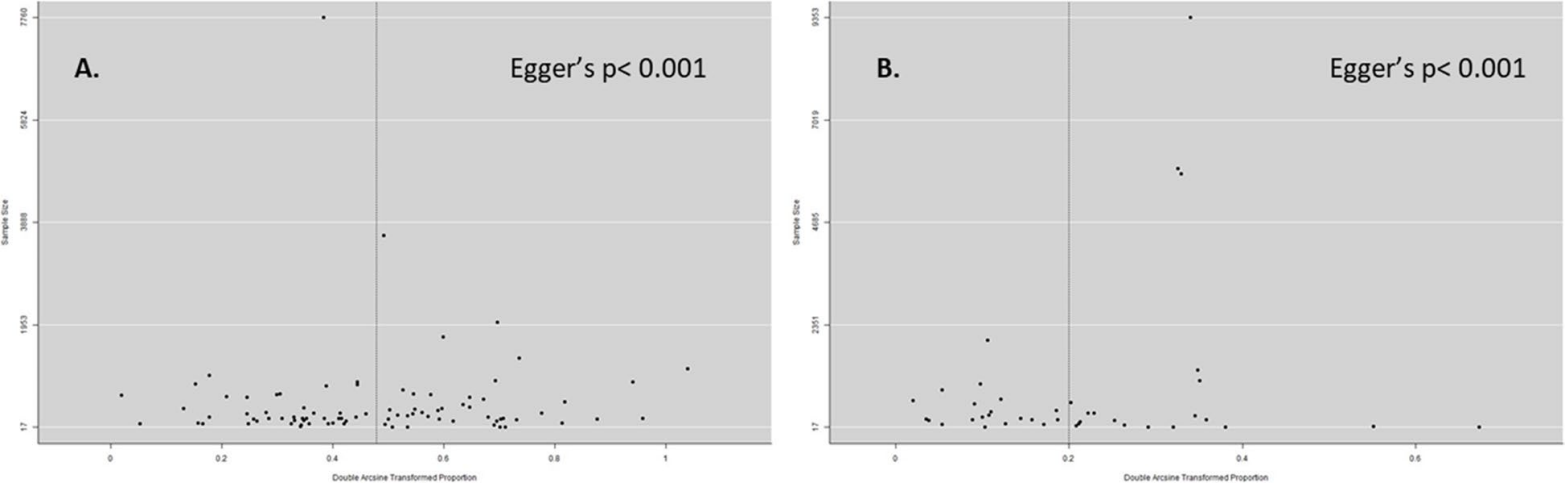
Funnel plot of anti-HEV IgG (**A**) and anti-HEV IgM prevalence. Double arcsine transformed proportion of individual studies is plotted against the sample size. The distribution of studies in the funnel plot revealed the presence of publication bias

We checked for publication bias using a funnel plot and Egger's test. Both the studies on anti-HEV IgG and IgM showed asymmetry with Egger's test indicating a *p*-value less than 0.001 for both cases (Fig. 6).

## Discussion

A paper search yielded varying numbers of manuscripts from Southeast Asian countries. The Philippines had the fewest studies, while Thailand had the highest with 15 studies. No data was found for Brunei Darussalam and East Timor or Timor Leste on the human species.

The results of this study provide valuable insights into the seroprevalence of IgG and IgM antibodies against HEV in different populations across Southeast Asian countries. Understanding the prevalence of these antibodies is essential for assessing the burden of HEV infection and identifying high-risk groups.

The extensive analysis of anti-HEV IgG prevalence in this study covered a wide range of population groups in Southeast Asia, including the general population, blood donors, pregnant women, hospital patients, farm workers, and chronic patients. The results unveiled an overall pooled prevalence of 21.03%, indicating significant exposure to the Hepatitis E virus among individuals in the region at some point in their lives. Moreover, a consistent increase in IgG prevalence was observed over the years, with the highest prevalence occurring in the most recent decade (2011–2023). This suggests a progressive rise in HEV exposure within the region.

Upon examining the prevalence data across different decades and population cohorts, a uniform upward trend in HEV antibody prevalence became apparent across all groups. Several factors could be assessed as potential contributors to this trend:

Notably, the expanding population in Southeast Asian nations during this timeframe increased the number of individuals at risk of Hepatitis E infection.

The rapid urbanization, characterized by the migration from rural to urban areas, led to higher population density and conditions conducive to Hepatitis E virus transmission [84]. Access to clean drinking water and adequate sanitation facilities emerged as critical factors in preventing Hepatitis E. Regions with inadequate infrastructure, particularly in water and sanitation, faced an elevated risk due to contaminated water sources. Climate-related events, such as heavy rainfall and flooding, significantly impacted waterborne diseases like Hepatitis E. The increasing frequency and severity of such events emphasized the importance of considering climate-related factors in assessing prevalence trends [85]. Consumption of contaminated or undercooked meat, particularly pork, was identified as a source of Hepatitis E transmission. Changes in food consumption habits over time may have contributed to changes in seroprevalence [86]. Limited access to healthcare facilities in certain areas exacerbated the spread of Hepatitis E. Increased awareness together with advances in medical research and the establishment of robust surveillance systems likely improved the detection and reporting of Hepatitis E cases, contributing to the observed increase in seroprevalence [87–89]. These multifaceted factors have likely played a collective role in shaping the changing landscape of Hepatitis E seroprevalence in Southeast Asian nations over the past decades. The upward trend emphasizes the importance of continued monitoring, intervention, and public health measures to mitigate the spread of Hepatitis E in the region.

Among specific populations, pregnant women exhibited an IgG prevalence of 18.56%, indicating that a considerable number of pregnant individuals have been exposed to HEV. Pregnant women are particularly vulnerable to the consequences of HEV infection, as it can lead to severe outcomes for both the mother and the foetus.

Hospital patients with clinical signs of acute infection showed an IgG prevalence of 16.3%, suggesting that HEV is still a significant cause of acute hepatitis cases in the hospital setting. Similarly, farm workers, especially those exposed to animals (reservoirs of HEV), had a high prevalence of IgG (28.4%), highlighting the occupational risk associated with zoonotic transmission.

Chronic patients, including individuals with chronic liver disease, HIV infection, or solid organ transplantation, exhibited the highest pooled IgG prevalence among all cohorts at 29.2%. This finding underscores the importance of monitoring HEV infection in immunocompromised individuals, as they may develop chronic HEV infection, which can lead to severe liver complications.

The prevalence of IgM antibodies, which are indicative of recent or acute HEV infection, was lower overall compared to IgG. The general population showed an IgM prevalence of 2.63% among acute infection cases. Among hospital patients exhibiting clinical signs of acute infection, the prevalence of IgM antibodies indicative of recent or acute HEV infection was higher at 4.45%.

Farm workers, particularly those exposed to animals, demonstrated the highest IgM prevalence at 6.21%. This finding highlights the occupational risk of acquiring acute HEV infection in this population due to direct or indirect contact with infected animals.

The study also identified a high-risk group, consisting of farm workers and chronic patients, with a pooled IgG prevalence of 28.9% and an IgM prevalence of 4.42%. This group is particularly susceptible to HEV infection and requires targeted interventions to reduce transmission and prevent severe outcomes.

Overall, this study provides valuable data on the seroprevalence of HEV antibodies in different populations in Southeast Asian countries. It highlights the importance of continued surveillance and public health interventions to control HEV transmission, especially in vulnerable groups. Understanding the prevalence trends over time can aid in developing effective strategies for the prevention and management of HEV infections in the region. However, further research and studies are warranted to explore the underlying factors contributing to the observed seroprevalence trends and to design targeted interventions to reduce HEV transmission in specific populations. Among the countries of Southeast Asia Myanmar was the most for HEV infection, while Malaysia registered the lowest seroprevalence.

This study has some limitations that we should be aware of. We looked at studies in three languages (English, Russian, and French), but we couldn't find data from two out of the 11 countries. This means we might not have a complete picture of the disease's prevalence in the whole region.

The way we divided the groups based on occupation or status could be questioned. Different criteria might give us different results, so it's something we need to consider. Another challenge is that the study covers a long time from 1989 to 2023 by published research and involves many different countries. This makes it difficult to compare the results because the tests used, and the diagnostic abilities might have changed over time and vary across countries.

Despite these limitations, our study presents a detailed epidemiologic report of combined seroprevalence data for HEV in Southeast Asian countries following the UN division. It gives us a basic understanding of the disease's prevalence in the region and offers some insights into potential risk factors. However, to get a more accurate picture, future research should address these limitations and include data from all countries in the region. Furthermore, certain countries such as Myanmar and the Philippines have not reported HEV prevalence data since 2006 and 2015, respectively. The absence of recent HEV prevalence reports from certain countries raises concerns about the availability of up-to-date epidemiological data for assessing the current status of hepatitis E virus infections in these regions.

## Conclusion

Our comprehensive analysis study involving Southeast Asian countries provides significant insights into the seroprevalence of hepatitis E virus (HEV) infection in this region and in various populations. The rates of anti-HEV antibodies observed among different groups, as well as the increasing trend in seroprevalence over decades, emphasize the dynamic nature of HEV transmission in the region. These findings contribute to a better understanding of HEV prevalence across countries, populations, and time periods in Southeast Asia, shedding light on important public health implications and suggesting directions for further research and intervention strategies.

### Supplementary Information

**Supplementary Material 1.**

**Supplementary Material 2.**

**Supplementary Material 3.**

## Data Availability

All data generated or analyzed during this study were included in this paper either in the results or supplementary information.

## Abbreviations

HEV: Hepatitis E Virus
PRISMA: Preferred reporting items for systematic review and meta-analysis
ELISA: Enzyme-Linked Immunosorbent Essay
HEV IgG: Hepatitis E virus Immunoglobulin G
HEV IgM: Hepatitis E Virus Immunoglobulin M
