# Supplementary material for: Systematic review and meta-analysis of hepatitis E seroprevalence in Southeast Asia: a comprehensive assessment of epidemiological patterns
Source: BMC Infect Dis. 2024 May 24;24:525. doi: 10.1186/s12879-024-09349-2 (PMC11127338; doi:10.1186/s12879-024-09349-2)
Supplement: Supplementary file 2 — Supplementary Material 2. [file 12879_2024_9349_MOESM2_ESM.docx]

Quality Assessment of Studies Included to the Study

JBI CRITICAL APPRAISAL CHECKLIST FOR STUDIES REPORTING PREVALENCE DATA (1)

Questions

1.         Was the sample frame appropriate to address the target population?

2.         Were study participants sampled in an appropriate way?

3.         Was the sample size adequate?

4.         Were the study subjects and the setting described in detail?

5.         Was the data analysis conducted with sufficient coverage of the identified sample?

6.         Were valid methods used for the identification of the condition?

7.         Was the condition measured in a standard, reliable way for all participants?

8.         Was there appropriate statistical analysis?

9.       Was the response rate adequate, and if not, was the low response rate managed appropriately?

|  | **Cambodia** | Ref. | Q1 | Q2 | Q3 | Q4 | Q5 | Q6 | Q7 | Q8 | Q9 |
| --- | --- | --- | --- | --- | --- | --- | --- | --- | --- | --- | --- |
| 1 | Kasper MR, 2012 | (33) | Y | Y | Y | Y | Y | Y | Y | Y | Y |
| 2 | Nouhin J, 2018 | (34) | Y | Y | Y | Y | Y | Y | Y | Y | Y |
| 3 | Nouhin J, 2016 | (35) | Y | Y | Y | Y | Y | Y | Y | Y | Y |
| 4 | Nouhin J, 2015 | (36) | Y | Y | Y | Y | Y | Y | Y | Y | Y |
| 5 | Yamada H, 2015 | (37) | Y | Y | Y | Y | Y | Y | Y | Y | Y |
| 6 | Chhour, YM, 2002 | (38) | Y | Y | Y | Y | Y | Y | Y | Y | Y |
| 7 | Buchy P, 2004 | (16) | Y | Y | N | Y | Y | Y | Y | Y | Y |
|  | | | | | | | | | | | |
|  | **Indonesia** |  | Q1 | Q2 | Q3 | Q4 | Q5 | Q6 | Q7 | Q8 | Q9 |
| 8 | Utsumi T, 2011 | (39) | Y | Y | Y | Y | Y | Y | Y | Y | Y |
| 9 | Achwan WA, 2007 | (41) | Y | Y | Y | Y | Y | Y | Y | Y | Y |
| 10 | Surya IG, 2005 | (42) | Y | Y | Y | Y | Y | Y | Y | Y | Y |
| 11 | Wibawa I D N, 2007 | (43) | Y | Y | Y | Y | Y | Y | Y | Y | Y |
| 12 | Sedyaningsih-Mamahit ER, 2002 | (28) | Y | Y | Y | Y | Y | Y | Y | Y | Y |
| 13 | Widasari DI, 2013 | (44) | Y | Y | Y | Y | Y | Y | Y | Y | Y |
| 14 | Corwin A, 1997 | (27) | Y | Y | Y | Y | Y | Y | Y | Y | Y |
| 15 | Corwin A, 1995 | (45) | Y | Y | Y | Y | Y | Y | Y | Y | Y |
| 16 | Wibawa ID, 2004 | (46) | Y | Y | Y | Y | Y | Y | Y | Y | Y |
| 17 | Jennings GB, 1994 | (14) | N | NA | Y | N | Y | Y | Y | Y | NA |
|  | | | | | | | | | | | |
|  | **Lao PDR** |  | Q1 | Q2 | Q3 | Q4 | Q5 | Q6 | Q7 | Q8 | Q9 |
| 18 | Bounlu K, 1998 | (48) | Y | Y | Y | Y | Y | Y | Y | Y | Y |
| 19 | Khounvisith V, 2023 | (49) | Y | Y | Y | Y | Y | Y | Y | Y | Y |
| 20 | Khounvisith V, 2018 | (50) | Y | Y | Y | Y | Y | Y | Y | Y | Y |
| 21 | Tritz SE, 2018 | (51) | Y | Y | Y | Y | Y | Y | Y | Y | Y |
| 22 | Bisayher S, 2019 | (24) | Y | Y | Y | Y | Y | Y | Y | Y | Y |
| 23 | Holt HR, 2016 | (52) | Y | Y | Y | Y | Y | Y | Y | Y | Y |
| 24 | Syhavong B, 2010 | (53) | Y | Y | Y | Y | Y | Y | Y | Y | Y |
| 25 | Chansamouth V, 2016 | (54) | Y | Y | Y | Y | Y | Y | Y | Y | Y |
| 26 | Pauly A, 2016 | (15) | Y | NA | Y | NA | NA | NA | Y | Y | NA |
|  | | | | | | | | | | | |
|  | **Malaysia** |  | Q1 | Q2 | Q3 | Q4 | Q5 | Q6 | Q7 | Q8 | Q9 |
| 27 | Wong LP, 2022 | (55) | Y | Y | NA | N | Y | Y | Y | Y | Y |
| 28 | Wong LP, 2022 | (56) | Y | Y | Y | Y | Y | Y | Y | Y | Y |
| 29 | Wong LP, 2020 | (57) | Y | Y | Y | Y | Y | Y | Y | Y | Y |
| 30 | Ng KP, 2000 | (20) | Y | Y | Y | Y | Y | Y | Y | Y | Y |
| 31 | Hudu, SA, 2018 | (58) | Y | Y | Y | n | Y | Y | Y | Y | Y |
| 32 | Seow HF, 1999 | (60) | NA | Y | N | N | Y | Y | Y | Y | Y |
| 33 | Saat Z, 1999 | (61) | Y | Y | Y | N | Y | Y | Y | Y | Y |
|  | | | | | | | | | | | |
|  | **Myanmar** |  | Q1 | Q2 | Q3 | Q4 | Q5 | Q6 | Q7 | Q8 | Q9 |
| 34 | Abe K, 2006 | (17) | Y | NA | Y | N | Y | Y | Y | Y | Y |
| 35 | Uchida T, 1993 | (30) | Y | Y | NA | Y | Y | Y | Y | Y | Y |
| 36 | Nakai K, 2001 | (63) | Y | Y | Y | Y | Y | Y | Y | Y | Y |
|  | | | | | | | | | | | |
|  | **Philippines** |  | Q1 | Q2 | Q3 | Q4 | Q5 | Q6 | Q7 | Q8 | Q9 |
| 37 | Lorenzo AA, 2015 | (17) | Y | NA | N | N | Y | Y | NA | Y | Y |
| 38 | Gloriani-Barzaga N, 1997 | (30) | Y | Y | N | Y | Y | Y | Y | Y | Y |
|  | | | | | | | | | | | |
|  | **Singapore** |  | Q1 | Q2 | Q3 | Q4 | Q5 | Q6 | Q7 | Q8 | Q9 |
| 39 | Chow WC, 1996 | (64) | Y | Y | Y | Y | Y | Y | Y | Y | Y |
| 40 | Wong CC, 2019 | (65) | Y | Y | Y | Y | Y | Y | Y | Y | Y |
| 41 | Tan LTC, 2013 | (66) | Y | N | Y | N | Y | Y | Y | Y | Y |
|  | | | | | | | | | | | |
|  | **Thailand** |  | Q1 | Q2 | Q3 | Q4 | Q5 | Q6 | Q7 | Q8 | Q9 |
| 42 | Pourpongporn P, 2009 | (21) | Y | Y | Y | Y | Y | Y | Y | Y | Y |
| 43 | Siripanyaphinyo U, 2014 | (67) | Y | Y | Y | Y | Y | Y | Y | Y | Y |
| 44 | Poovorawan Y, 2016 | (68) | Y | Y | Y | Y | Y | Y | Y | Y | Y |
| 45 | Maneerat Y, 1996 | (22) | N | N | N | N | Y | Y | Y | Y | NA |
| 46 | Sa-nguanmoo P, 2015 | (69) | Y | Y | Y | Y | Y | Y | Y | Y | Y |
| 47 | Pilakasiri C, 2009 | (23) | Y | Y | Y | Y | Y | Y | Y | Y | Y |
| 48 | Louisirirotchanakul S, 2002 | (70) | Y | Y | NA | Y | Y | Y | Y | Y | Y |
| 49 | Jupattanasin S, 2019 | (71) | Y | Y | Y | Y | Y | Y | Y | Y | Y |
| 50 | Hinjoy S, 2013 | (72) | Y | Y | Y | Y | Y | Y | Y | Y | Y |
| 51 | Getsuwan S, 2023 | (73) | Y | Y | Y | Y | Y | Y | Y | Y | Y |
| 52 | Gonwong S, 2014 | (74) | Y | Y | Y | Y | Y | Y | Y | Y | Y |
| 53 | Komolmit P, 2020 | (75) | Y | Y | Y | Y | Y | Y | Y | Y | Y |
| 54 | Jutavijittum, P., 2000 | (76) | Y | Y | Y | Y | Y | Y | Y | Y | Y |
| 55 | Boonyai A, 2021 | (77) | Y | Y | Y | Y | Y | Y | Y | Y | Y |
| 56 | Abe K, 2006 | (17) | Y | NA | Y | Y | Y | Y | Y | Y | Y |
|  | | | | | | | | | | | |
|  | **Vietnam** |  | Q1 | Q2 | Q3 | Q4 | Q5 | Q6 | Q7 | Q8 | Q9 |
| 57 | Huy PX, 2021 | (78) | Y | Y | Y | Y | Y | Y | Y | Y | Y |
| 58 | Ostankova, Yu.V., 2021 | (19) | Y | Y | Y | Y | Y | Y | Y | Y | Y |
| 59 | Hoan NX, 2019 | (79) | Y | Y | Y | Y | Y | Y | Y | Y | Y |
| 60 | Hoan NX, 2015 | (80) | Y | Y | Y | Y | Y | Y | Y | Y | Y |
| 61 | Lichnaia E.V, 2021 | (18) | Y | Y | Y | Y | Y | Y | Y | Y | Y |
| 62 | Hau CH, 1999 | (81) | Y | Y | Y | Y | Y | Y | Y | Y | Y |
| 63 | Corwin AL, 1996 | (29) | Y | Y | Y | Y | Y | Y | Y | Y | Y |
| 64 | Corwin AL, 1996 | (82) | Y | Y | Y | Y | Y | Y | Y | Y | Y |
| 65 | Berto A, 2016 | (83) | Y | Y | Y | Y | Y | Y | Y | Y | Y |
| 66 | Abe K, 2006 | (17) | Y | NA | Y | N | Y | Y | Y | Y | Y |
| 67 | Shimizu K, 2016 | (85) | Y | NA | Y | N | Y | Y | Y | Y | Y |
| 68 | Nghiem XH, 2018 | (25) | Y | N | Y | N | NA | NA | Y | Y | Y |
| 69 | Tran HTT, 2003 | (86) | Y | Y | Y | N | N | Y | Y | Y | N |
| 70 | Buchy P, 2004 | (16) | Y | Y | N | Y | Y | Y | Y | Y | Y |

1. JOANNA BRIGGS INSTITUTE 2017. The Joanna Briggs Institute Critical Appraisal tools for use in JBI systematic reviews–checklist for case series. 2016.
