# Supplementary material for: Systematic review and meta-analysis of hepatitis E seroprevalence in Southeast Asia: a comprehensive assessment of epidemiological patterns
Source: BMC Infect Dis. 2024 May 24;24:525. doi: 10.1186/s12879-024-09349-2 (PMC11127338; doi:10.1186/s12879-024-09349-2)
Supplement: Supplementary file 3 — Supplementary Material 3. [file 12879_2024_9349_MOESM3_ESM.pptx]

## Slide 1
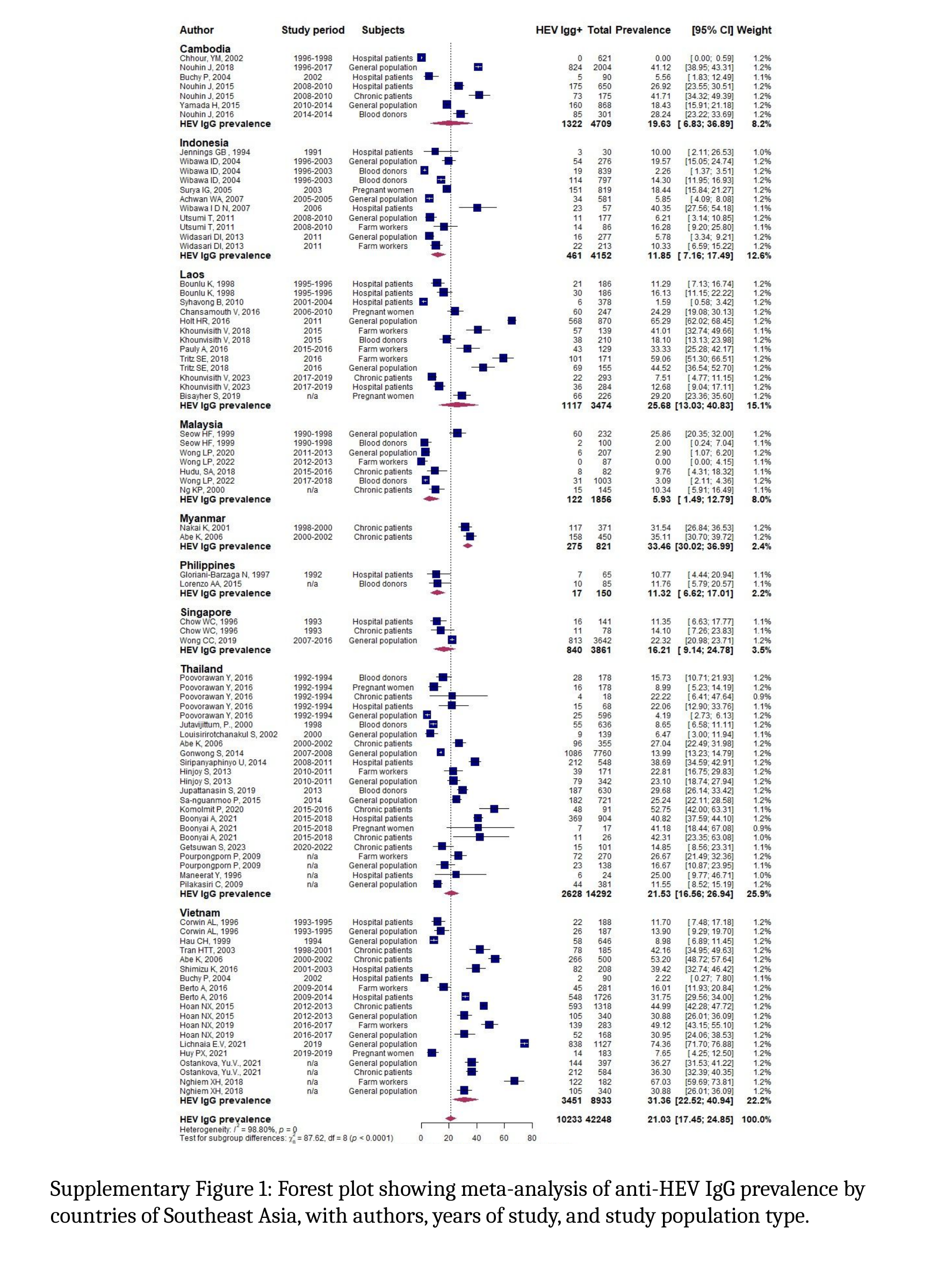

Supplementary Figure 1: Forest plot showing meta-analysis of anti-HEV IgG prevalence by countries of Southeast Asia, with authors, years of study, and study population type.

## Slide 2
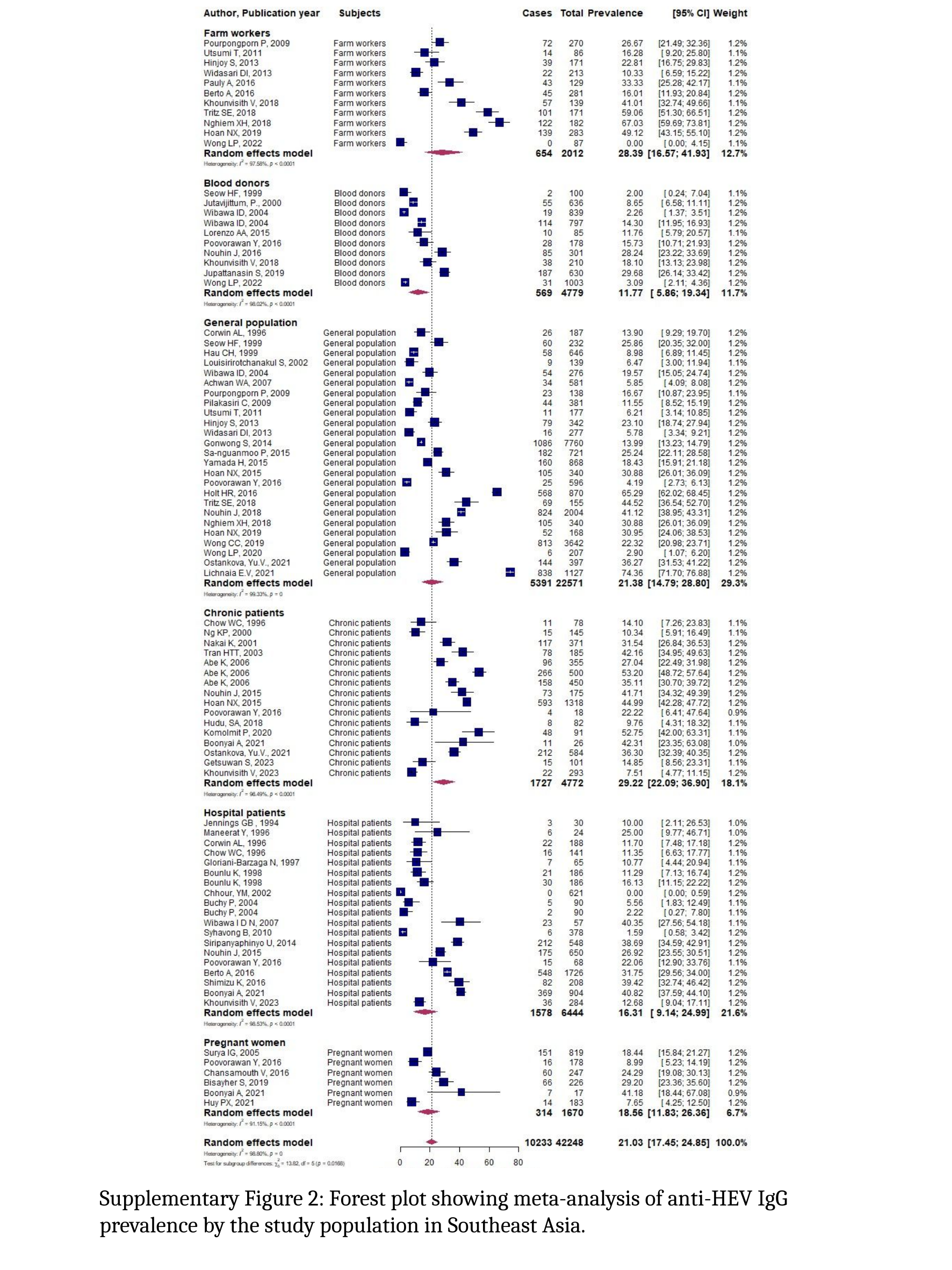

Supplementary Figure 2: Forest plot showing meta-analysis of anti-HEV IgG prevalence by the study population in Southeast Asia.

## Slide 3
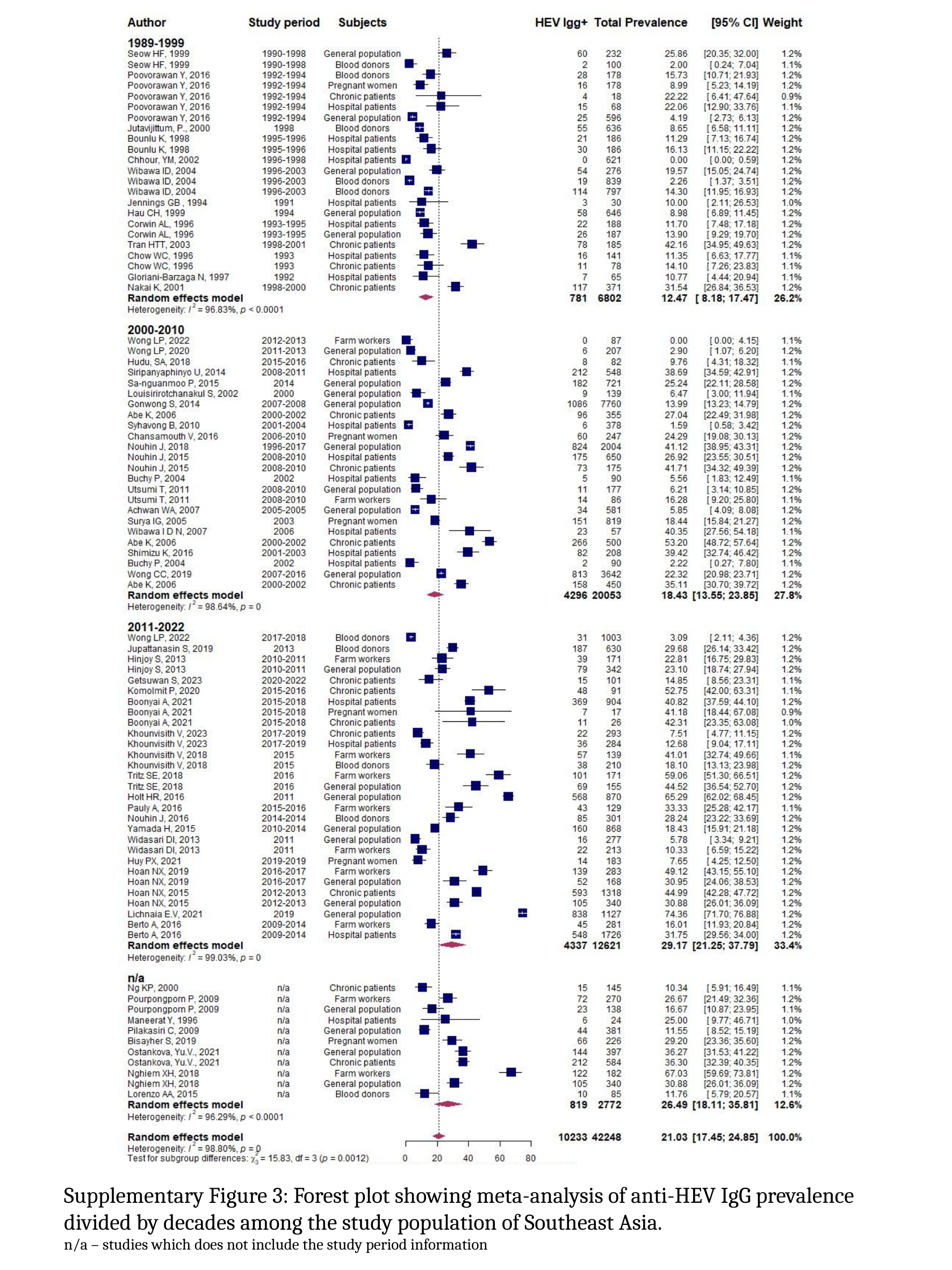

Supplementary Figure 3: Forest plot showing meta-analysis of anti-HEV IgG prevalence divided by decades among the study population of Southeast Asia.
n/a – studies which does not include the study period information

## Slide 4
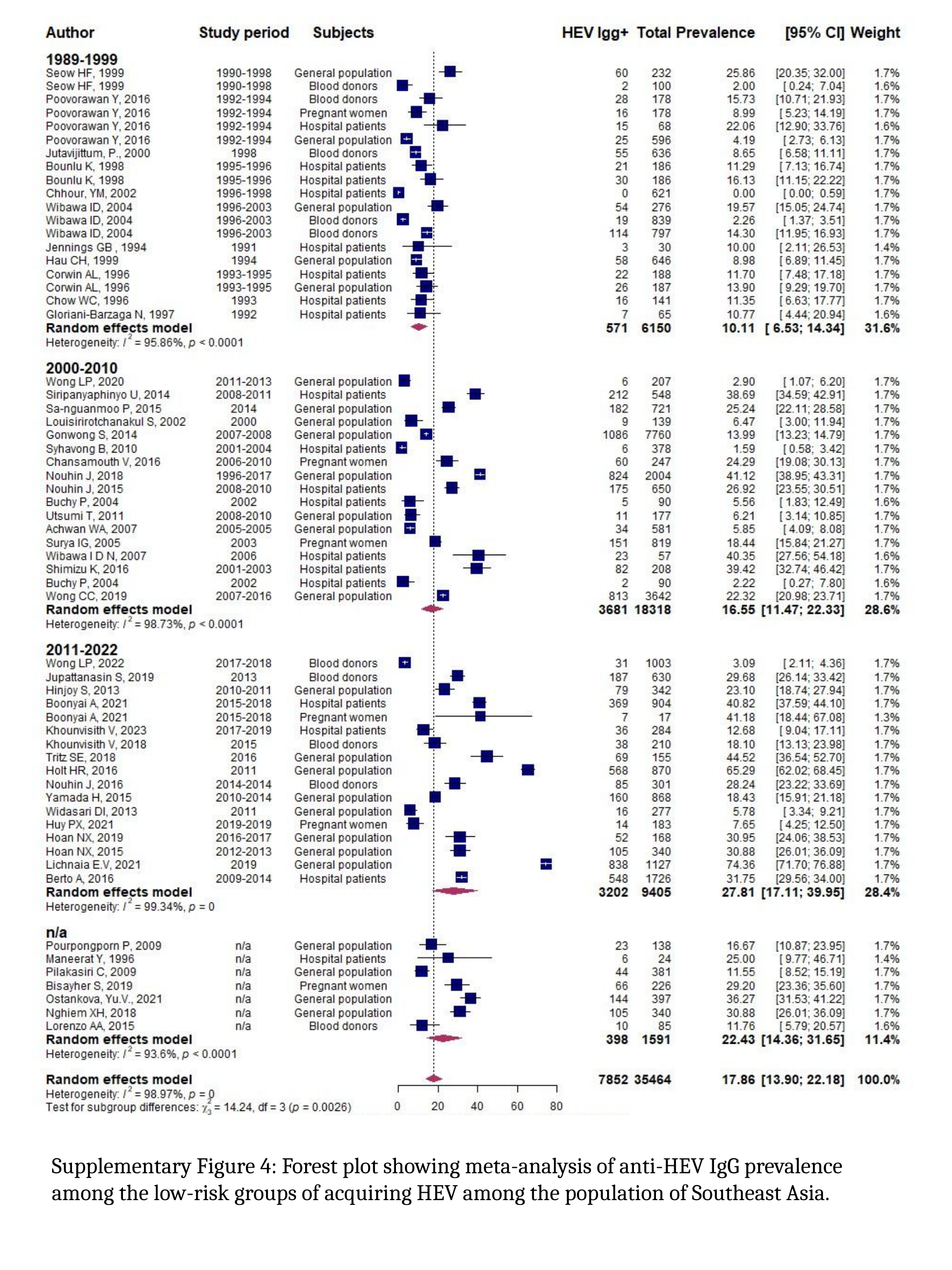

Supplementary Figure 4: Forest plot showing meta-analysis of anti-HEV IgG prevalence among the low-risk groups of acquiring HEV among the population of Southeast Asia.

## Slide 5
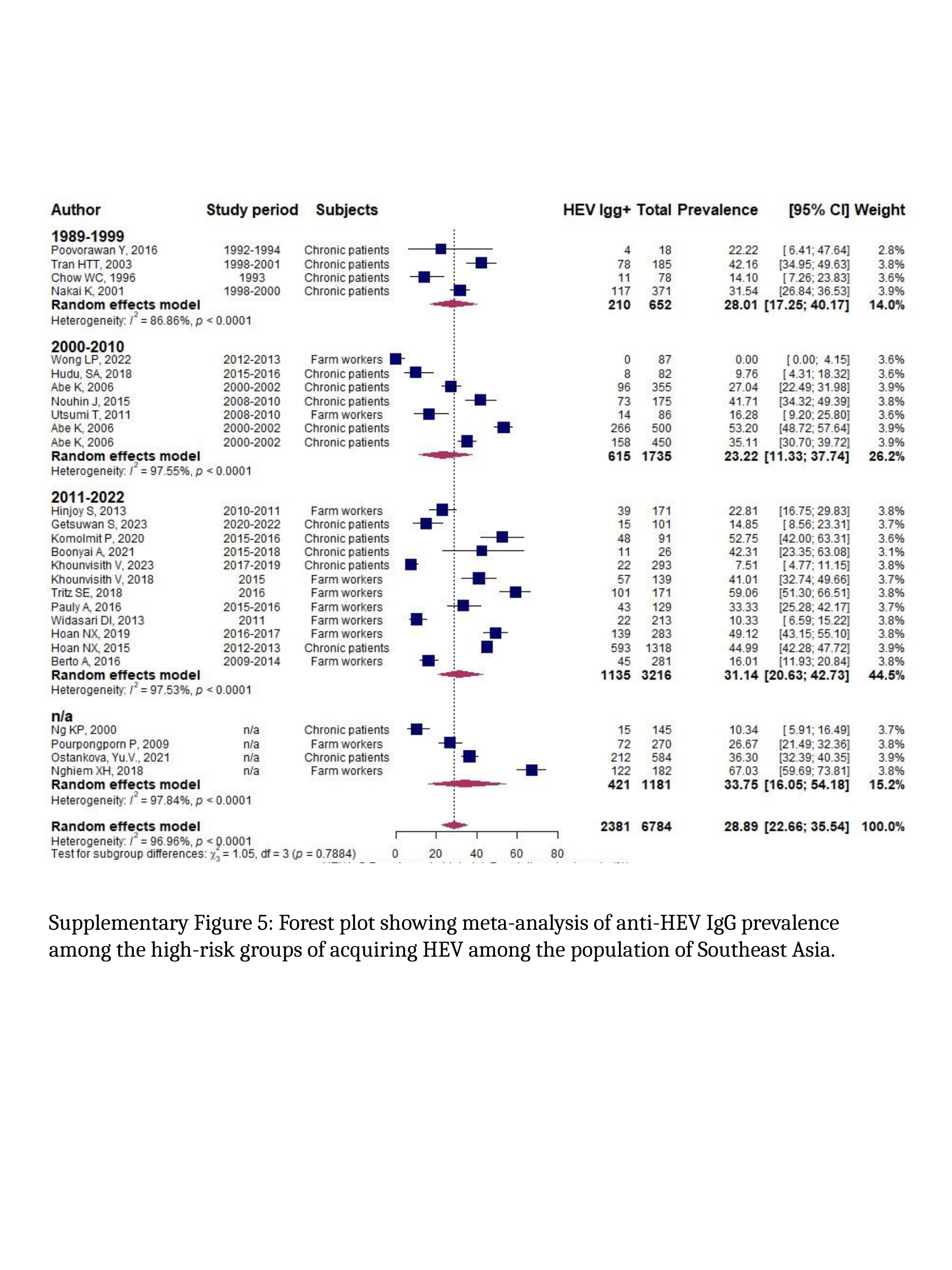

Supplementary Figure 5: Forest plot showing meta-analysis of anti-HEV IgG prevalence among the high-risk groups of acquiring HEV among the population of Southeast Asia.

## Slide 6
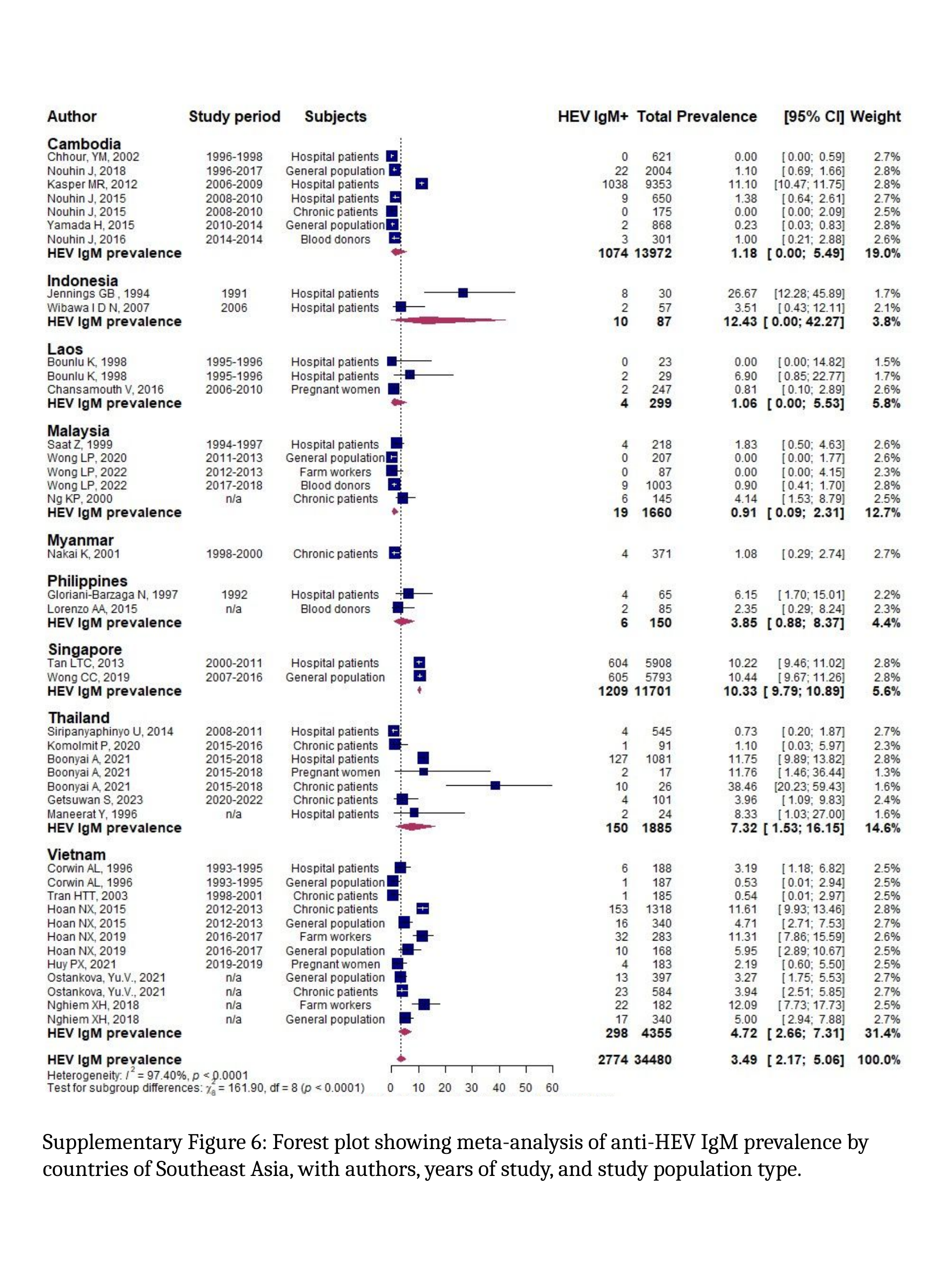

Supplementary Figure 6: Forest plot showing meta-analysis of anti-HEV IgM prevalence by countries of Southeast Asia, with authors, years of study, and study population type.

## Slide 7
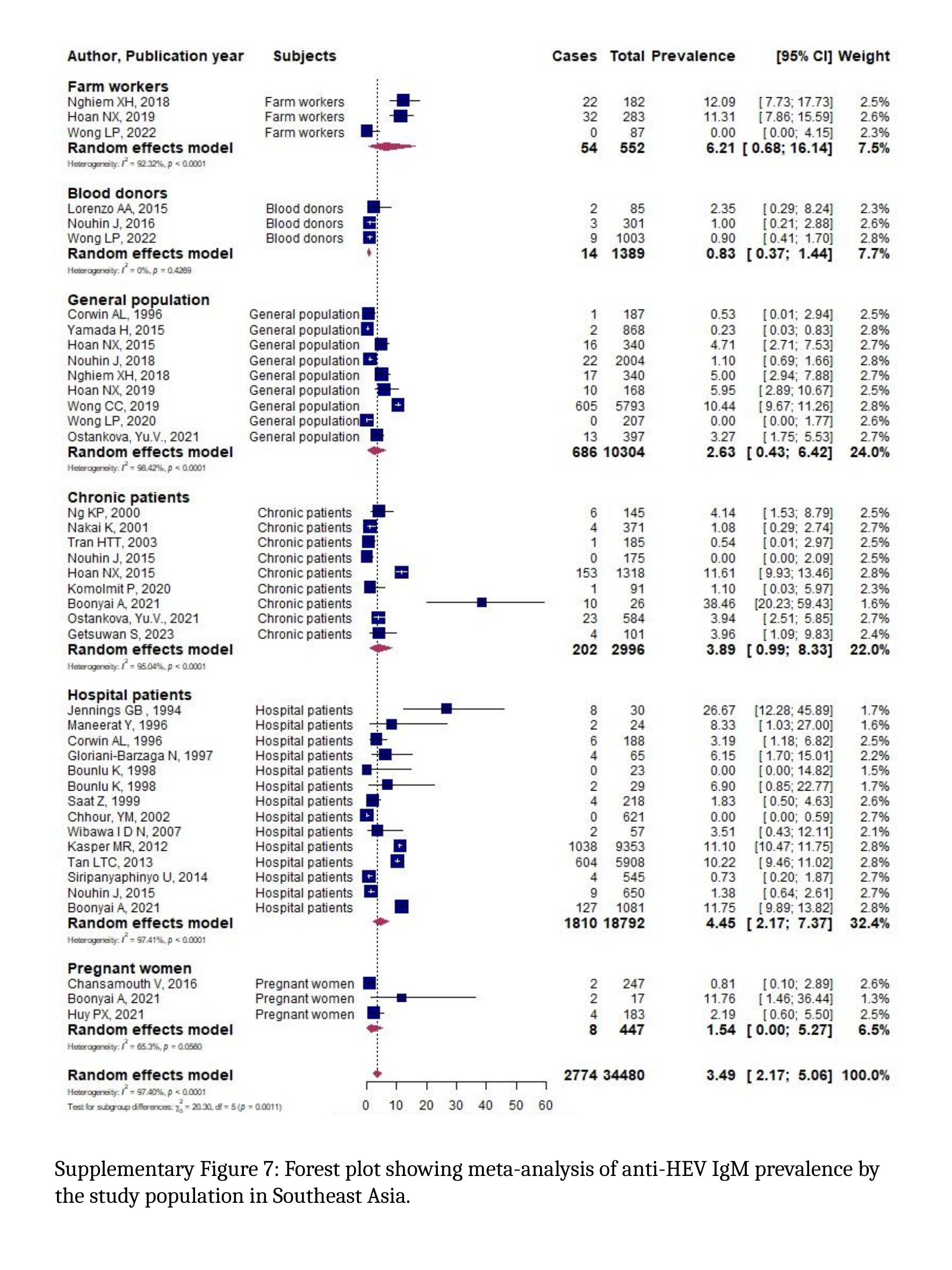

Supplementary Figure 7: Forest plot showing meta-analysis of anti-HEV IgM prevalence by the study population in Southeast Asia.

## Slide 8
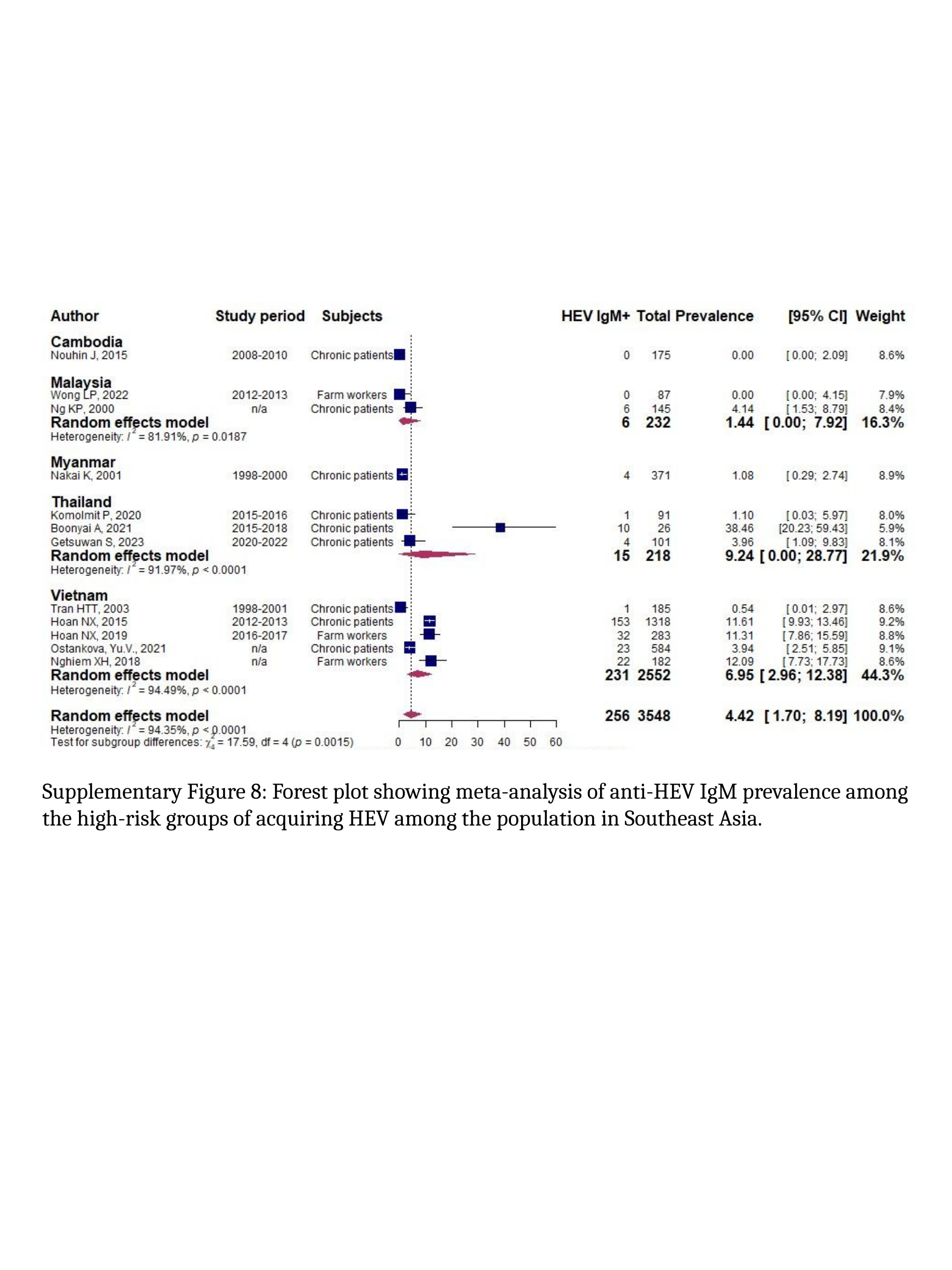

Supplementary Figure 8: Forest plot showing meta-analysis of anti-HEV IgM prevalence among the high-risk groups of acquiring HEV among the population in Southeast Asia.

## Slide 9
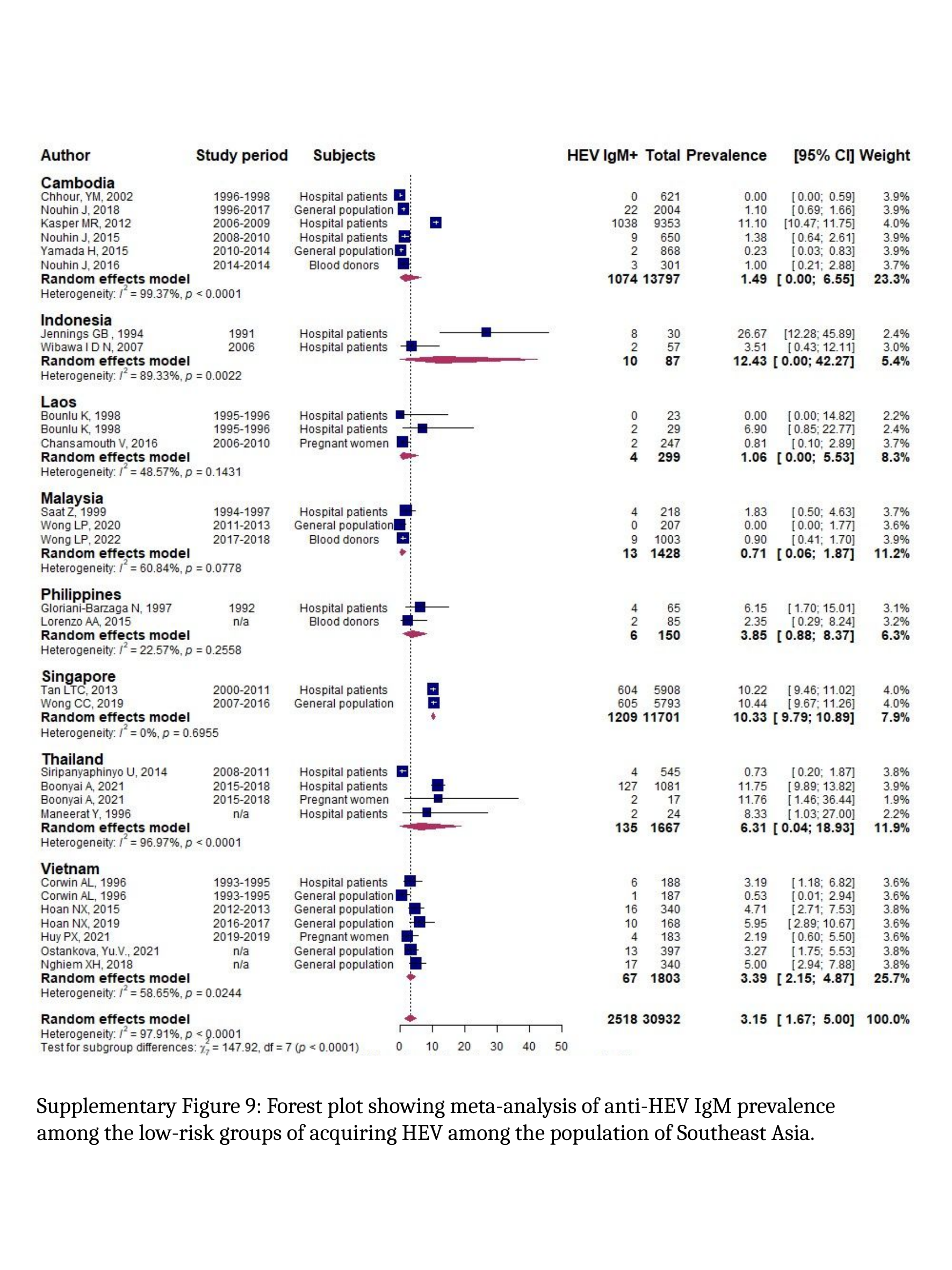

Supplementary Figure 9: Forest plot showing meta-analysis of anti-HEV IgM prevalence among the low-risk groups of acquiring HEV among the population of Southeast Asia.

## Slide 10
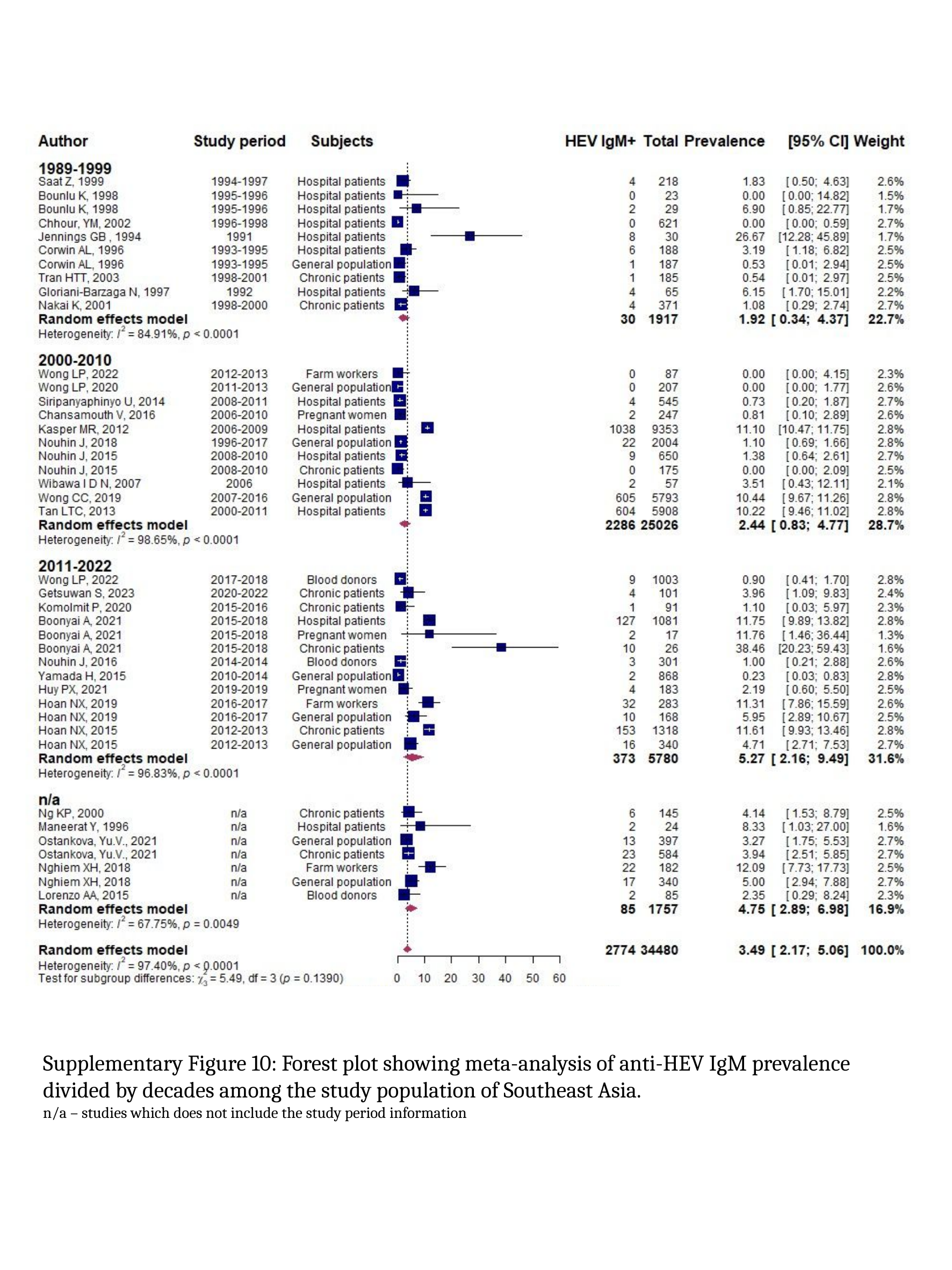

Supplementary Figure 10: Forest plot showing meta-analysis of anti-HEV IgM prevalence divided by decades among the study population of Southeast Asia.
n/a – studies which does not include the study period information

## Slide 11
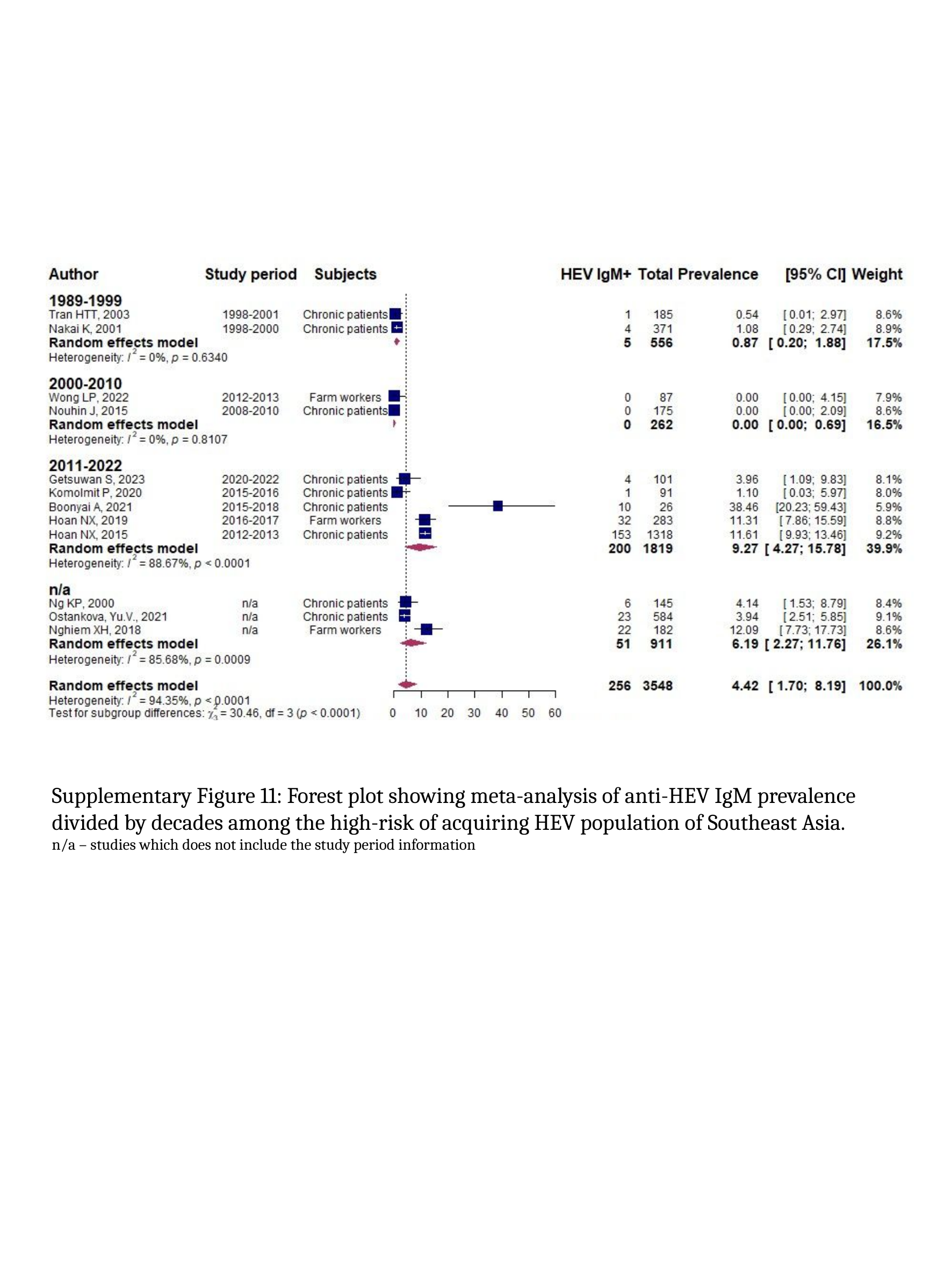

Supplementary Figure 11: Forest plot showing meta-analysis of anti-HEV IgM prevalence divided by decades among the high-risk of acquiring HEV population of Southeast Asia.
n/a – studies which does not include the study period information

## Slide 12
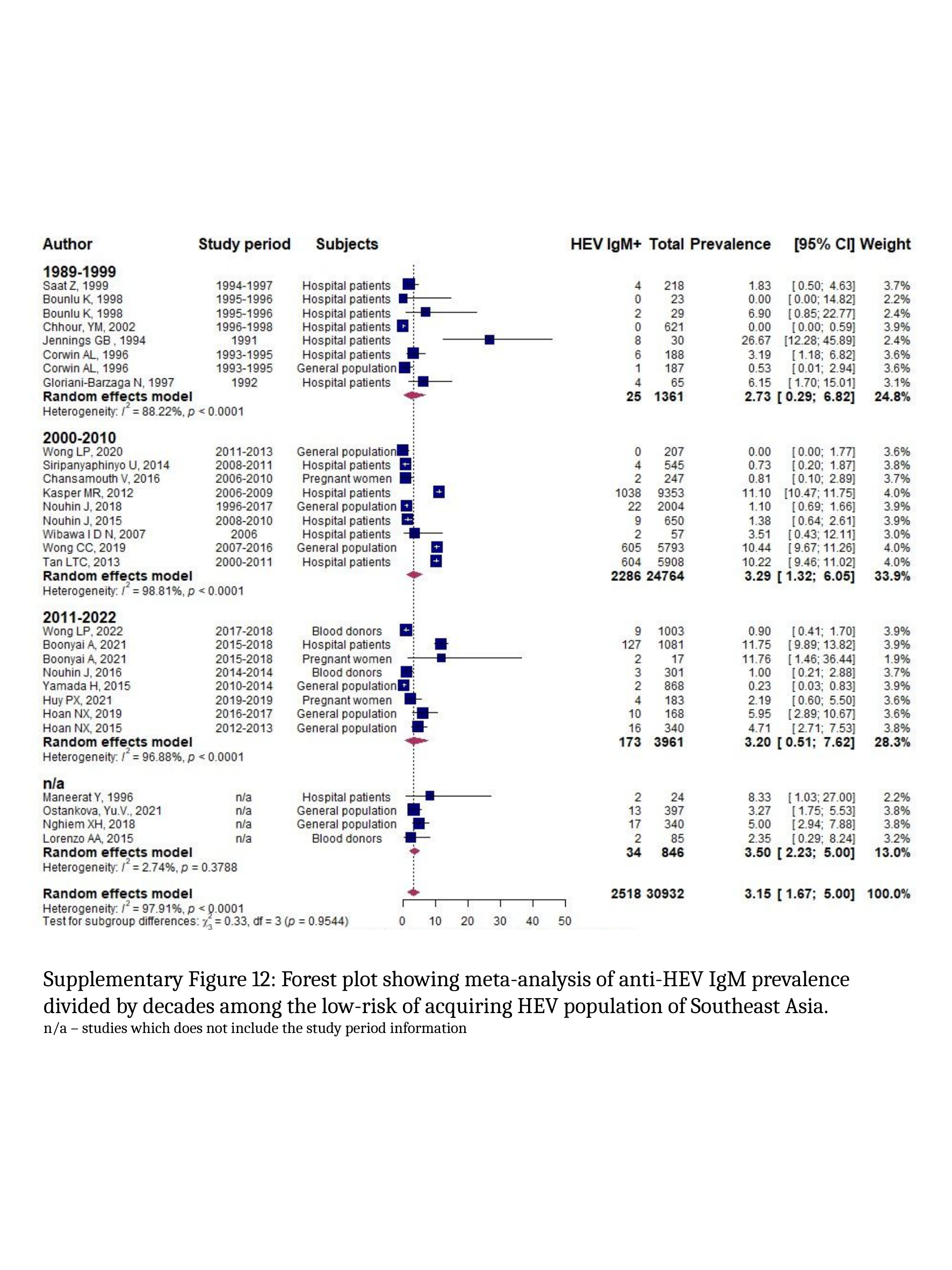

Supplementary Figure 12: Forest plot showing meta-analysis of anti-HEV IgM prevalence divided by decades among the low-risk of acquiring HEV population of Southeast Asia.
n/a – studies which does not include the study period information

## Slide 13
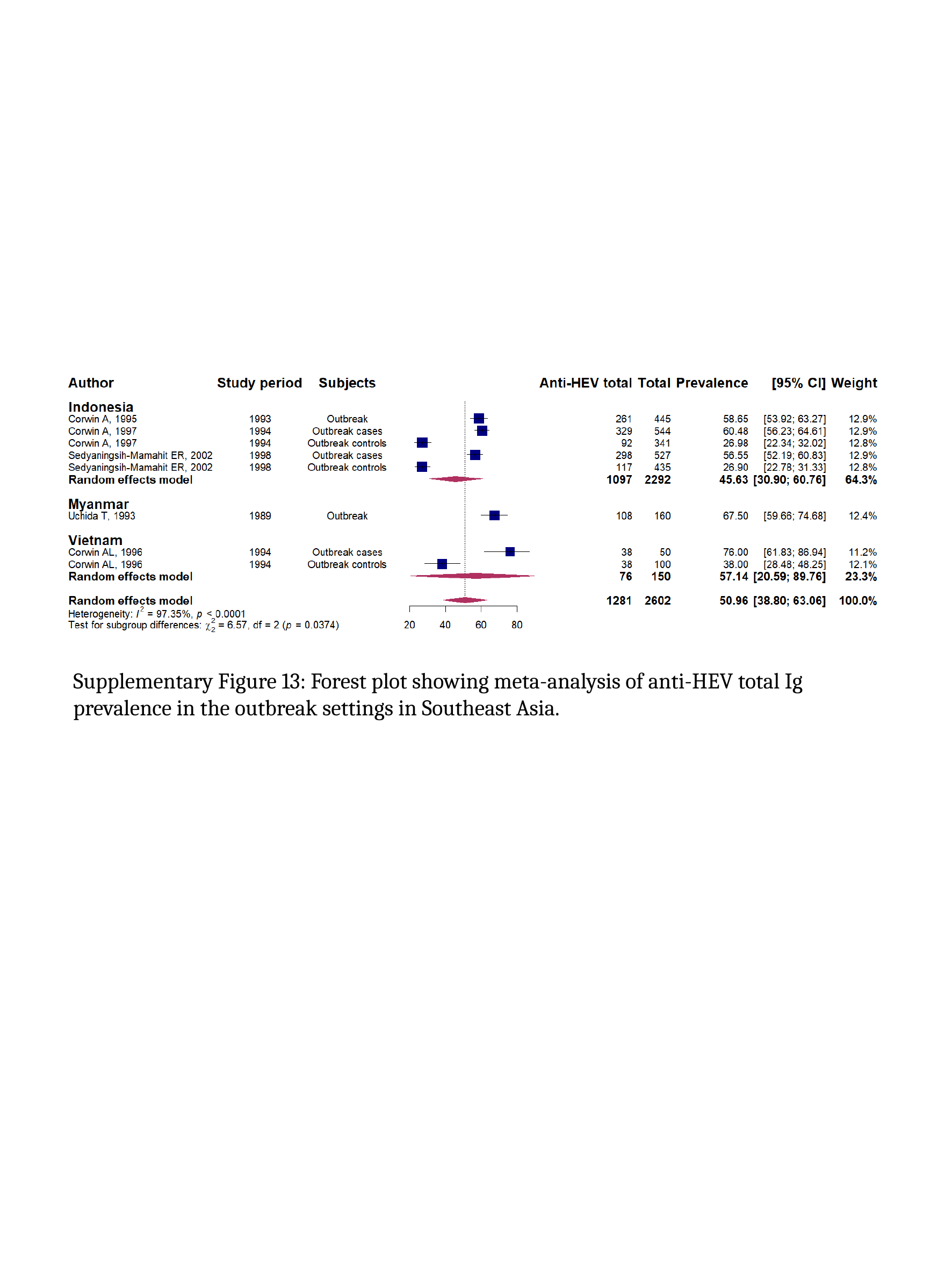

Supplementary Figure 13: Forest plot showing meta-analysis of anti-HEV total Ig prevalence in the outbreak settings in Southeast Asia.
